# Comparative Genomics Reveals Evolutionary Traits, Mating Strategies, and Pathogenicity-Related Genes Variation of *Botryosphaeriaceae*

**DOI:** 10.3389/fmicb.2022.800981

**Published:** 2022-02-23

**Authors:** Chengming Yu, Yufei Diao, Quan Lu, Jiaping Zhao, Shengnan Cui, Xiong Xiong, Anna Lu, Xingyao Zhang, Huixiang Liu

**Affiliations:** ^1^Shandong Research Center for Forestry Harmful Biological Control Engineering and Technology, College of Plant Protection, Shandong Agricultural University, Taian, China; ^2^Research Institute of Forest Ecology, Environment and Protection, Chinese Academy of Forestry, Beijing, China; ^3^Institute of Forestry New Technology, Chinese Academy of Forestry, Beijing, China

**Keywords:** *Botryosphaeriaceae*, phytopathogen, evolution traits, mating strategies, pathogenicity-related genes

## Abstract

*Botryosphaeriaceae*, as a major family of the largest class of kingdom fungi *Dothideomycetes*, encompasses phytopathogens, saprobes, and endophytes. Many members of this family are opportunistic phytopathogens with a wide host range and worldwide geographical distribution, and can infect many economically important plants, including food crops and raw material plants for biofuel production. To date, however, little is known about the family evolutionary characterization, mating strategies, and pathogenicity-related genes variation from a comparative genome perspective. Here, we conducted a large-scale whole-genome comparison of 271 *Dothideomycetes*, including 19 species in *Botryosphaeriaceae*. The comparative genome analysis provided a clear classification of *Botryosphaeriaceae* in *Dothideomycetes* and indicated that the evolution of lifestyle within *Dothideomycetes* underwent four major transitions from non-phytopathogenic to phytopathogenic. Mating strategies analysis demonstrated that at least 3 transitions were found within *Botryosphaeriaceae* from heterothallism to homothallism. Additionally, pathogenicity-related genes contents in different genera varied greatly, indicative of genus-lineage expansion within *Botryosphaeriaceae*. These findings shed new light on evolutionary traits, mating strategies and pathogenicity-related genes variation of *Botryosphaeriaceae*.

## Introduction

*Dothideomycetes* represents the largest and most important class of ascomycete fungi, including 23 orders, 110 families, 1,261 genera, and 19,000 species (Wijayawardene et al., 2017). The members of *Dothideomycetes* comprise both phytopathogenic (Ohm et al., 2012) and non-phytopathogenic fungi with diverse lifestyles (Ruibal et al., 2009) as well as many mycorrhizal fungi (Peter et al., 2016). Among these 110 families, *Botryosphaeriaceae* is an important and distinctive family. This is because it includes saprobes, endophytes, and phytopathogens, and it is one of the most widely geographically distributed groups of opportunistic plant pathogens. The host range of this family is very wide, and many economically important plants worldwide can be infected by them (Slippers and Wingfield, 2007). These pathogenic fungi can infect plants through wounds or natural openings, such as lenticels and stomata. Once they enter host tissues, they may survive as endophytes to stay at a biotrophic stage for a long time and turn into the destructive necrotrophic stage when the host is stressed (Yan et al., 2013; Morales-Cruz et al., 2015). The members of *Botryosphaeriaceae* can infect many woody plants and cause serious disease symptoms, such as dieback, branch canker, leaf spots, and fruit and seed rot (Marsberg et al., 2017). But the interaction of some of *Botryosphaeriaceae* fungi like *Botryosphaeria dothidea* (*B. dothidea*) with host plants includes a latent or endophytic phase, which makes the fungal infection easily be neglected (Bostock et al., 2014; Marsberg et al., 2017; Wang et al., 2018). Therefore, it is of substantial biological significance to explore the evolutionary characteristics of *Botryosphaeriaceae* at the level of *Dothideomycetes*.

The exceptional feature of *Botryosphaeriaceae* fungi is that it is difficult to observe their sexual structure under both natural and experimental conditions, but this does not mean that sexual reproduction does not occur for these fungi (Phillips et al., 2013). With more in-depth research being conducted, the mating strategies (homothallism or heterothallism) of increasing members of the *Botryosphaeriaceae* family have been reported (Bihon et al., 2012a,b, 2014; Billiard et al., 2012; Lopes et al., 2017, 2018), revealing some unresolved questions regarding their sexual reproduction. For example, how conservative are the nucleic acid and protein sequences of the mating type determination genes; how conservative are the genes and their arrangement at the mating type determination loci; how has the mating type evolved, and what is the origin type. In addition, the host range of different *Botryosphaeriaceae* fungi varies greatly. *B. dothidea* is a common pathogen with a wide range of hosts. Generally, the infection becomes symptomatic when the host is subjected to drought, physical damage, waterlogging, or freezing stress (Bostock et al., 2014; Marsberg et al., 2017; Wang et al., 2018). The symptoms primarily include canker on young seedlings, branches, and stems; the necrosis of branches; and fruit decay, which may lead to the death of the host in extreme cases (Kim et al., 2005; Tang et al., 2012). *Botryosphaeria kuwatsukai* (*B. kuwatsukai*) can also cause symptoms, such as fruit softening and decay, and severe canker of branches and stems (Wang et al., 2021). However, *B. kuwatsukai* has a relatively narrow host range and primarily infects apple and pear trees (Xu et al., 2015). Therefore, a systematic study of the mating strategies and differences of pathogenicity-related genes in *Botryosphaeriaceae* fungi will lead to a better understanding of their molecular evolutionary history and pathogenic characteristics.

Currently, genomics technology has been widely used to study many pathogenic fungi of plants, and greatly promoted the understanding of their evolution and pathogenic mechanisms (Islam et al., 2012; Blanco-Ulate et al., 2013; van der Nest et al., 2014; Nagel et al., 2018; Félix et al., 2019; Landi et al., 2020; Meile et al., 2020). For example, gene family expansion associated with virulence factors in wood-colonizing pathogenic fungi in the *Botryosphaeriaceae* was revealed via phylogenomic comparisons (Garcia et al., 2021); the comparative genome analyses of latent plant pathogens in the *Botryosphaeriaceae* were conducted to define their genomes (Nagel et al., 2021). In addition, a large-scale comparative genomic analysis can better reveal the physiological characteristics and evolutionary history of fungi (Guttman et al., 2014; Haridas et al., 2020; Miyauchi et al., 2020). Therefore, in this study, 167 *Dothideomycetes* fungi, including 19 *Botryosphaeriaceae* species, were fully sequenced, and a comparative genomics approach was used to comprehensively analyze the molecular evolution characteristics, mating strategies and pathogenicity within *Botryosphaeriaceae* fungi. The sequence differences of related genes benefit our understanding of the evolutionary history of *Botryosphaeriaceae* fungi and provide useful information for the prevention and control of the diseases caused by these fungi.

## Materials and Methods

### Fungal Strains

A total of 167 *Dothideomycetes* fungal strains, including 160 *Botryosphaeriaceae* fungi (19 species) (Supplementary Table 1), from the Fungal Strain Library of Shandong Agricultural University, Tai’an, China, were sequenced in this study. The sequencing data are found at NCBI (PRJNA777748).

### Sequencing and Genome Assembly

The CTAB (hexadecyltrimethylammonium bromide) method (Murray and Thompson, 1980) was used to extract high-quality DNA. A 500 bp DNA fragment library was constructed, and PE150 sequencing was performed using Illumina HiSeq 4,000 (San Diego, CA, United States). The raw data obtained from sequencing were inputted into Trimmomatic v0.39 (Bolger et al., 2014) for quality control, and reads with an average quality of less than 30 were filtered. Jellyfish v2.3.0 (Marçais and Kingsford, 2011) was used to calculate k-mer distribution and GenomeScope v2.0 (Ranallo-Benavidez et al., 2020) was then used to assess genome size of each fungus. The assembly was carried out using SPAdes v3.13.1 (Bankevich et al., 2012).

### Gene Prediction and Genome Annotation

RepeatModeler v2.0.1^1^ was used to construct custome repeat libraries for each assembly, and RepeatMasker v4.1.1^2^ was used to determine repeat contents with the custom repeat libraries. For *ab initio* gene prediction, GeneMark-ES v4.48_3.60 (Lomsadze et al., 2014) and AUGUSTUS v3.2.1 (Stanke and Morgenstern, 2005) were used with default parameters. First, GeneMark-ES was used to predict gene models, then the models were used to train AUGUSTUS. Exonerate v2.2.0 (Slater and Birney, 2005) was used for homology comparison prediction. Finally, MAKER v3.01.03 (Campbell et al., 2014) was used to predict protein-coding genes by combining the gene models from GeneMark-ES, AUGUSTUS, and Exonerate. BUSCO v4.1.4 was used to evaluate the completeness of genomes and genome annotations based on pezizomycotina_odb9 (3156 core ortholog genes) (Waterhouse et al., 2018). Functional annotations on the putative genes were performed using the following softwares: BLAST v2.8.1 + (Camacho et al., 2009) for the NCBI non-redundant protein (NR, 2020-06), SwissProt and FunSecKB2 databases (E-value threshold of 1E-05); HMMER v3.2.1 (Eddy, 2008) for the Pfam 32.0 and TransportDB 2.0 databases; KofamKOALA v1.2.0 (Aramaki et al., 2020) for KEGG annotation; and dbCAN2 (Zhang et al., 2018) for annotating carbohydrate active enzymes (CAZy).

### Evolutionary Analysis of *Botryosphaeriaceae* Fungi

To construct an accurate evolutionary tree, eight outgroups (Supplementary Table 1) were selected and OrthoFinder v2.3.11 (Emms and Kelly, 2015) was used for gene family clustering. According to the clustering results, single copy orthogroups were extracted and aligned using MAFFT v7.427 (Katoh et al., 2002). These processed single copy orthogroups were then concatenated using a self-written Perl script and filtered using Gblock v0.91b (Castresana, 2000). PartitiosnFinder v2.1.1 (Lanfear et al., 2017) and RAxML v.8.2.2 (Stamatakis, 2014) were used to determine the optimal amino acid substitution model and build the Maximum Likelihood (ML) tree with 1,000 bootstrap replicates, respectively.

The known ecologies states (multistate and binary) and FUNGuild were used to determine the ecologies of each species (Nguyen et al., 2016). Mesquite v3.61 software was then used to infer ancestral ecological character states (Haridas et al., 2020).

### Functional Enrichment Analysis of *Botryosphaeriaceae*

To find the differences in gene function annotation of *Botryosphaeriaceae* fungi with different lifestyles, scipy.stats package in Python was used to perform the two-tailed Fisher exact test for functional annotation with *p*-value threshold of 0.01. To reduce false positives, functional annotations with the number of genes less than 100 were filtered.

### Mating Strategies of *Botryosphaeriaceae*

#### Mating Type Genes and Surrounding Genes

To determine the presence of mating type genes in the *Botryosphaeriaceae* fungal genome, the mating type genes of *Diplodia sapinea* (*D. sapinea*) and some neighboring genes (KF551229 and KF551228) (Bihon et al., 2012a) were used as templates to search homologous genes from the putative *Botryosphaeriaceae* fungal genes that has been generated in this study, using the partial alignment mode in BLASTx (Camacho et al., 2009). The mating type genes obtained were then inputted into the NCBI’s conserved domain database (Marchler-Bauer et al., 2015) to determine their functional domains.

#### Comparison of the Arrangement of Mating Type Loci

To compare the arrangement of mating type genes and their surrounding genes in the *Botryosphaeriaceae* fungal genome, a BLASTn alignment was conducted and then EasyFig version 2.2.2 (Sullivan et al., 2011) was used for collinearity analysis, with the *E* value threshold set to 1e^–4^.

#### Phylogenetic Comparison and Ancestral State Reconstruction

To study the mating strategies of *Botryosphaeriaceae* fungi, ML evolutionary trees of 24 *Botryosphaeriaceae* fungi were constructed with single-copy genes (using the same method described in section: Comparison of the Arrangement of Mating Type Loci). Briefly, OrthoFinder v2.3.11 (Emms and Kelly, 2015) was used to cluster gene families and extract single copy orthogroups. Each orthogroup was aligned and concatenated with MAFFT v7.427 (Katoh et al., 2002). Gblock v0.91b (Castresana, 2000) was used for filtering, and finally, RAxML v.8.2.2 (Stamatakis, 2014) was used to build an ML tree with the LG + I + G + F model. Mesquite v3.61^3^ and the Mk1 likelihood model were used to reconstruct the evolutionary process of homothallism (hom) and heterothallism (het).

#### Changes in Genes Related to the Pathogenicity of *Botryosphaeriaceae* Fungi

The production of phytotoxic compounds in pathogenic fungi, such as secondary metabolite, secreted proteins, and carbohydrate-active enzymes, is one of the important infective weapons (Amselem et al., 2011). The genomes of *Botryosphaeriaceae* were searched for genes encoding the phytotoxic compounds and then the number difference of these genes was statistically analyzed as previously described (Wang et al., 2018).

Specifically, 23 *Botryosphaeriaceae* fungi (7 genera) and 10 other representative fungi were included to analyze changes in genes related to the pathogenicity in *Botryosphaeriaceae*. The 10 representative fungi contained 1 biotrophic fungus (*Puccinia graminis*), 2 necrotrophic fungi (*Valsa mali* and *Pyrenophora triticirepentis*), 2 saprophytic fungi (*Neurospora crassa* and *Rhizopus oryzae*), 3 hemibiotrophic fungi (*Pyricularia oryzae*, *Colletotrichum higginsianum*, and *Zymoseptoria tritici*), 1 symbiotic fungus (*Laccaria bicolor*), and 1 endophytic fungus (*Peltaster fructicola*).

## Results

### Genome Sequencing, Assemby, and Annotation

In this study, 167 genomes of *Dothideomycetes*, including 160 *Botryosphaeriaceae* (19 species), were sequenced, assembled, and annotated (Supplementary Table 1). All genomes were sequenced at average coverage 166 ± 48 x. The average assembled genome lengths of 167 *Dothideomycetes* ranged between 28.85 and 61.78 Mb, which were consistent with the sizes estimated by k-mer counting approach. The contig N50 values of assembled genomes varied from 41.81 to 779.02 kb with a mean of 241.77 kb. The repeat contents of assembled genomes varied from 0.95 to 10.98% with a mean of 5.18%. All assembled genomes have a high completeness with an average of 94.8 ± 4.3%, and the similar result was also found in genome annotations (average of 98.0 ± 2.9%).

### Classification Based on Whole-Genome Data

**FIGURE 1:**
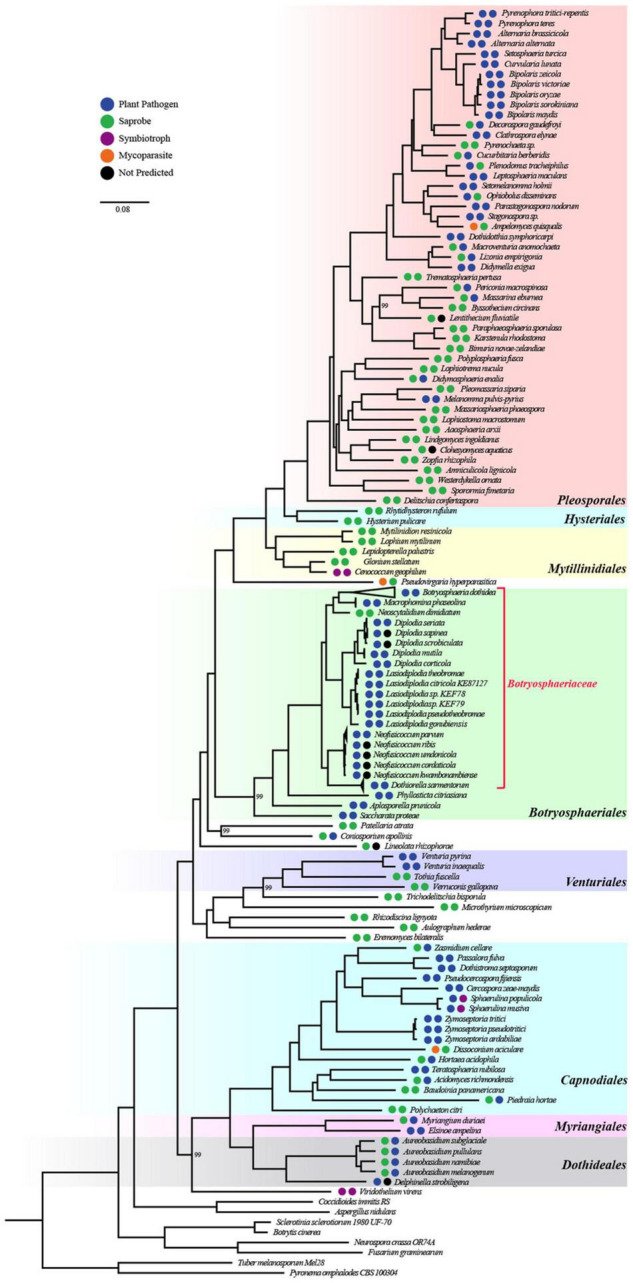
Whole-genome-based phylogenetic tree of 271 species from *Dothideomycetes* and 8 outgroups. All bootstrap values are 100% except for those shown. The orders of *Dothideomycetes* were well classified and were displayed by different colors. The two circles left of species names standed for lifestyle classification according to organism data and FunGuild, respectively.

To better understand the evolutionary characteristics of *Botryosphaeriaceae*, we used 167 newly sequenced in this study and 112 genomes available in public database to construct a whole-genome phylogenetic tree using 480 single-copy gene families. The phylogenetic tree was clearly divided into 2 clades, corresponding to 2 subclasses, namely *Pleosporomycetidae* and *Dothideomycetidae* (Supplementary Figure 1). A comprehensive phylogenetic analysis revealed that the members of *Botryosphaeriaceae* that belonged to *Botryosphaeriales* were added to *Pleosporomycetidae* (Figure 1 and Supplementary Table 2).

### Saprophytic Fungi Have a Larger Genome Size Than Phytopathogenic Fungi

The genome sizes of *Dothideomycetes* ranged from 17 (*Piedraia hortae*) to 177 Mbp (*Cenococcum geophilum*), and 7,896–34,881 protein-coding genes were detected (Supplementary Figures 2, 3 and Supplementary Table 1). Compared with non-pathogenic fungi, pathogenic fungi usually have a smaller genome (Supplementary Figure 2). The genome size of *Botryosphaeriaceae* is in the range of 28.85 (*Aureobasidium pullulans*) −61.78 Mbp (*Dothiorella sarmentorum*) (47.19 Mbp on average), and 11,505–16,851 proteion-coding genes were predicted (13,765 genes in average).

### Saprophytic Fungi Are the Possible Evolutionary Ancestors of *Botryosphaeriaceae* Fungi

**FIGURE 2:**
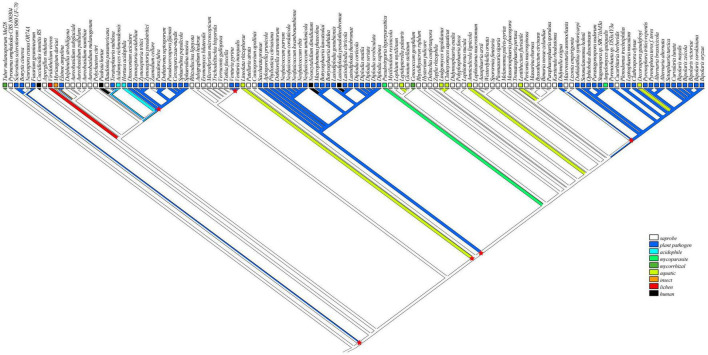
Reconstruction of ancestral lifestyle character state of *Dothideomycetes* using Mesquite based on parsimony model as saprobe. Major lifestyle shifts were marked by six red star symbols.

To infer the ecological characteristics of ancestors of *Botryosphaeriaceae* fungi, we analyzed their lifestyle evolution process at the class level. The results showed that the ancestral lifestyle was likely to be the saprophytic type (Figure 2), which is also supported by the maximum likelihood analysis (Supplementary Figure 4 and Supplementary Table 3). During the evolution of *Dothideomycetes* fungi, at least 6 transitions from non-phytopathogenic (NPP) to phytopathogenic (PP) were detected, including 4 major transitions, which are presented at the MRCA node of *Mycosphaerellaceae* (*Dothideomycetidae*) (Node 225: NPP = 0.1868, PP = 0.8132), *Venturia* (*Pleosporomycetidae*) (Node 189: NPP = 0.0039, PP = 0.9961) and *Botryosphaeriales* (Node 20: NPP = 0.0984, PP = 0.9016), along with the branching point of *Setomelanomma*-*Bipolaris* (Node 98: NPP = 0.0074, PP = 0.9926). In addition, the *Botryosphaeriaceae* fungi have undergone at least 3 transitions from saprophytic to pathogenic fungi, including Node 176 (NPP = 0.9859, PP = 0.0141), Node 180 (NPP = 0.9992, PP = 0.0008) and Node 246 (NPP = 0.9101, PP = 0.0899) (Supplementary Table 3).

### Differences in the Gene Families of Plant Pathogenic and Saprophytic Fungi

To investigate the differences between plant pathogenic and saprophytic fungi from the perspective of gene family contractions and expansions, we used Fisher’s exact test to perform these analyses on gene families. Differences in 78 Pfam and 58 GO annotations were found between saprophytic and plant pathogenic fungi. In the saprophytic fungi, 42 Pfam and 35 GO terms showed more than 20% of expansion, and 31 Pfam and 14 GO terms displayed more than 20% of contraction. In plant pathogenic fungi, 32 Pfam and 17 GO terms showed greater than 20% of expansion, and 35 Pfam and 21 terms displayed more than 20% of contraction. Compared with saprophytic fungi, the plant pathogenic fungi contained more gene families that showed contraction. Compared with saprophytic fungi, 94 Pfam and 67 GO terms of *Botryosphaeriaceae* fungi showed greater than 20% of expansion, while 52 Pfam and 44 GO terms displayed contractions (> 20%) (Supplementary Table 4).

### Mating Strategies of *Botryosphaeriaceae* Fungi

#### Mating Type Genes

To determine the mating type genes, *MAT1-1* and *MAT1-2* of 24 fungi (7 genera and 19 species) were analyzed in the *Botryosphaeriaceae* family. The results showed that the *Botryosphaeria*, *Neofusicoccum*, and *Dothiorella* genomes harbored both *MAT1-1* and *MAT1-2*, indicating that they were homothallic. *Diplodia*, *Macrophomina*, and *Neoscytalidium* genomes harbored either *MAT1-1* or *MAT1-2*, indicating that they were heterothallic fungi. *Lasiodiplodia* fungi had various mating strategies, including homothallism (*L. gonubiensis*) and heterothallism (*Lasiodiplodia citricola*, *L. pseudotheobromae*, and *L. theobromae*) (Supplementary Table 5). In addition, the protein domains of MAT1-1-1 and MAT1-2-1 were highly conservative. All the MAT1-1-1 proteins contain MATalpha domains, and all the MAT1-2-1 proteins contain MAT_HMG-box domains (Supplementary Table 6). Compared with the neighboring genes, the nucleic acid sequences of the mating type genes were poorly conserved (Supplementary Table 7), while the length of the coding sequence and the position and size of the intron were largely conservative.

#### Arrangement of Mating Loci

**FIGURE 3:**
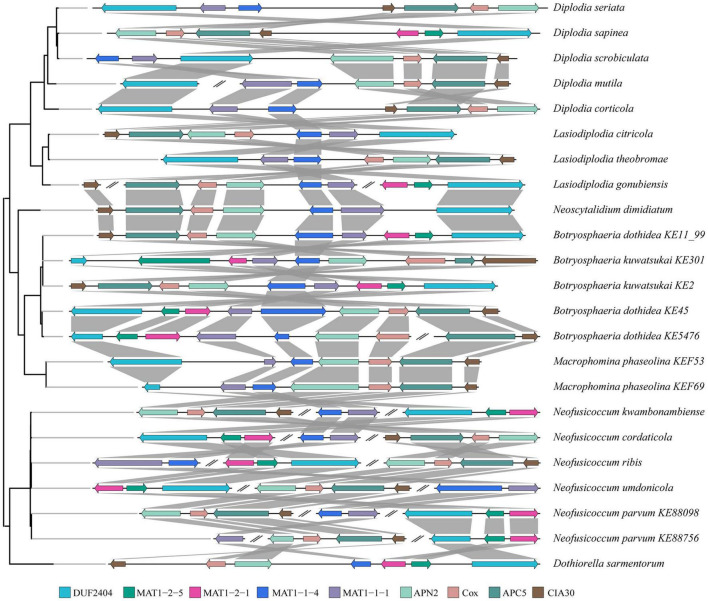
Pairwise mating type and surrounding genes comparison between species of *Botryosphaeriaceae*. Genes (color coded arrows) were on genomic sequences (horizontal lines). Organization of genes were indicated by gray box. Abbreviations of genes: putative integral membrane protein containing DUF2404 domain (DUF2404), DNA lyase (APN2), cytochrome C oxidase subunit Via (Cox), anaphase-promoting complex subunit 5 (APC5) and Complex I intermediate-associated protein 30 (CIA30).

Arrangement analysis of mating loci showed that there were three types of arrangements at the mating type determining loci of *Botryosphaeriaceae* (Figure 3). In most *Botryosphaeriaceae* genomes (e.g., *Diplodia sapinea*), the *MAT1* gene was primarily located between a collinear region that contains 4 protein-coding genes and 1 putative integral membrane (PIM) protein (Figure 3). The PIM contains pleckstrin homology domains and DUF2404. The four genes in the collinear region encode, in order, a DNA lyase (APN2), cytochrome c oxidase subunit VIa (CoxVIa), anaphase-promoting complex subunit 5 (APC5), and complex I intermediate-associated protein 30 (CIA30). According to the positional relation between the *MAT1* and *APN2* genes (the *MAT1* gene was located at the proximal end of APN2), the second type of arrangement was observed in *Botryosphaeria*, *L. gonubiensis* (*Lasiodiplodia*), *M. phaseolina* (*Macrophomina*), and *N. dimidiatum* (*Neoscytalidium*). For example, the *MAT1* gene of *M. phaseolina* and *B. dothidea* was located upstream of the *APN2* gene, while in *L. gonubiensis* and *N. dimidiatum*, the *MAT1* gene was located downstream of the *APN2* gene, and the four genes in the collinear region were arranged in reverse order. However, the opposite arrangement was observed in the two strains of *B. kuwatsukai*-the *MAT1* gene of the PG2 strain was located downstream of the *APN2* gene, while LW030101 was located upstream. A similar type of reversed arrangement as the one found for *B. kuwatsukai* was also identified in two *B. dothidea* strains. The third type of arrangement was primarily observed for *Neofusicoccum* species in which the two subtypes of *MAT1*, *MAT1-1*, and *MAT1-2*, were not located in conjunction; they were either located both distantly from the collinear region or at different chromosomes (or scaffolds) (Figure 3).

#### Reconstruction of the Ancestral State of the Mating Strategy

To understand the evolutionary characteristics of the mating type of *Botryosphaeriaceae*, we selected 24 representative *Botryosphaeriaceae* strains to reconstruct the evolutionary process of homothallism and heterothallism mating strategies in *Botryosphaeriaceae* (Supplementary Figure 5). Our analysis showed that het mating was likely to be the ancestral type (Node 2, het = 0.6198, hom = 0.3802) (Supplementary Figure 5 and Supplementary Table 8). During the course of evolution, *Botryosphaeriaceae* fungi have undergone at least 2 major transitions to their hom strategy. The first was located at the branching point of *Lasiodiplodia theobromae* and *L. citricola* (Node 14: het = 0.8291, hom = 0.1709), and the other transition was primarily observed at the branching point of *Neofusicoccum* and *Dothiorella* (Node 26: het = 0.4344, hom = 0.5656) (Supplementary Figure 5 and Supplementary Table 8).

#### Changes in Genes Related to the Pathogenicity of Botryosphaeriaceae

##### Secondary Metabolism

**FIGURE 4:**
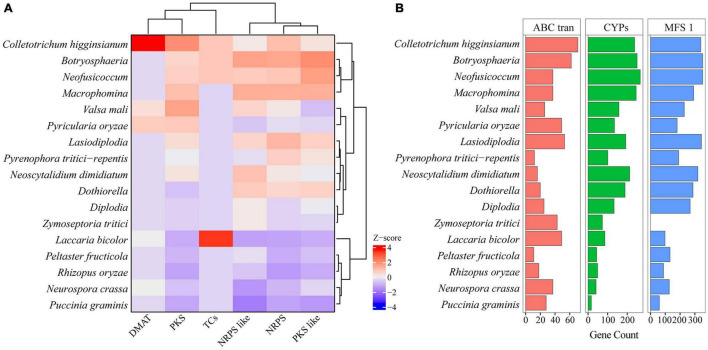
Comparing backbone and related genes of secondary metabolism among *Botryosphaeriaceae* fungi and other 10 fungal species. **(A)** Key gene family of secondary metabolism. In each column, Z-score was used to describe the trend of over-represented (+ 4–0) and down-represented (0 to −4) gene family. **(B)** Gene family number comparison of MFS_1, ABC_tran and CYPs among *Botryosphaeriaceae* fungi and other 10 fungal species.

To find the key enzymes involved in the synthesis of secondary metabolites in *Botryosphaeriaceae* fungi, we used Pfam annotations to find 3 types of genes that encode these key enzymes, such as polyketide synthase (PKS), non-ribosomal peptide synthetase (NRPS), and dimethylallyl tryptophane synthase (DMATS). The PKS genes of *Botryosphaeriaceae* fungi differed significantly from each other (*p* = 2.0 × 10^–16^) (Figure 4A). Among all the species, the *Macrophomina* fungi contained the largest number of PKS genes (31 on average), while the *Dothiorella* fungi contained the least number (8 on average) (Supplementary Table 9). The genes related to secondary metabolites in fungi also included those that encode cytochrome P450 enzymes, regulatory factors, and transporters. Cytochromes P450 can catalyze the transformation of hydrophobic intermediates in the primary and secondary metabolic pathways and plays an important role in fungi. Compared with other fungi, *Botryosphaeriaceae* fungi contained the largest number of genes that encode cytochromes P450 (133-267). Among all the species, *Neofusicoccum* fungi contained the highest number of cytochromes P450 (267), followed by *Botryosphaeria* (*n* = 251) (Figure 4B). The *ATP-binding cassette* (*ABC*) or *major facilitator superfamily* (*MFS*) gene families played important roles in the transport of secondary metabolites. The numbers of *ABC* or *MFS* in *Botryosphaeriaceae* fungi were higher than those of other species (*ABC*: 268-353; *MFS*: 16-62). Among all the species, *Botryosphaeria* fungi contained the largest number of *MFS* (*n* = 62), while *Neoscytalidium dimidiatum* fungi contain the least amount (*n* = 16) (Figure 4B).

##### Secreted Proteins

**FIGURE 5:**
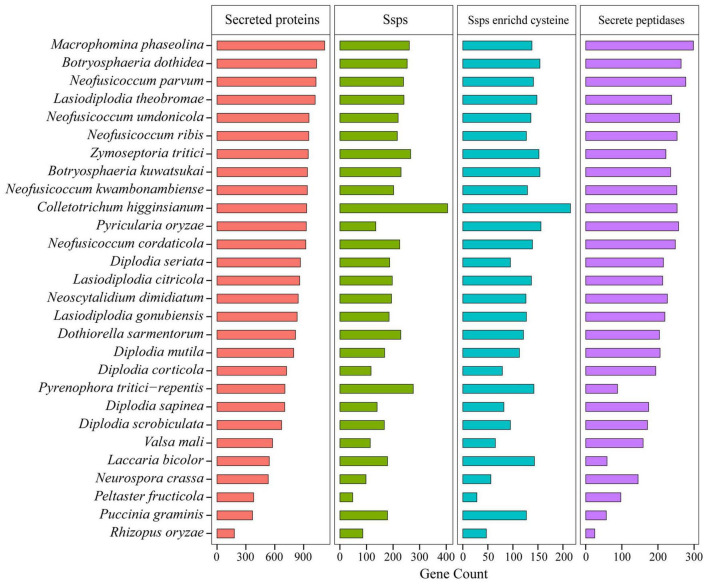
Comparison of secreted proteins among *Botryosphaeriaceae* fungi and other 10 fungal species.

Pathogens can secrete a battery of proteins, which are deployed to the host-pathogen interface during infection, and these secreted proteins played important roles in fungal pathogenicity (O’Connell et al., 2012; Fernandes et al., 2014; Félix et al., 2016). In this study, we predicted the secreted proteins of *Botryosphaeriaceae* (Figure 5). The number of secreted proteins in *Botryosphaeria* (the *B. dothidea* fungi contain 1,034, and the *B. kuwatsukai* fungi contain 939) was not significantly different from *Macrophomina* (*n* = 1,118), and the *B. kuwatsukai* fungi seemed to secrete less proteins. The number of proteins secreted by the *Neofusicoccum* fungi varied greatly (922–1,028). Among all the species, the *N. parvum* fungi contained the largest number (*n* = 1,028), and *N. cordaticola* fungi contained the least (*n* = 922). Compared with other fungal genera, the *Diplodia* genus contained fewer secreted proteins (671–866) (Figure 5 and Supplementary Table 10).

The transfer of secreted effector proteins to host plant cells was the key to pathogenesis of many plant pathogenic microorganisms. Secreted proteins less than 200 amino acids in length and rich in cysteine were considered as candidate secreted effectors (Wang et al., 2018). The *Botryosphaeriaceae* family contained two genera *Macrophomina* and *Botryosphaeria* that may release a large number of secreted proteins, and the number of small, secreted proteins of these two genera was also higher than that of other genera. For example, *M. phaseolina* (261), *B. dothidea* (253), and *B. kuwatsukai* (230) displayed significant expansions of gene families. Small secretory proteins rich in cysteine were the most abundant in *Botryosphaeria* fungi and the least in *Diplodia* fungi (79-113) (Figure 5 and Supplementary Table 10). In *Botryosphaeria*, both *B. dothidea* and *B. kuwatsukai* contained 154 small secretory proteins rich in cysteine, and the Pfam annotations of them included 19 and 21 known functional domains, respectively (Supplementary Table 11). By comparing the Pfam annotations of *B. dothidea* and *B. kuwatsukai* secretory proteins, we found that ribonuclease, the cerato-platanin family, and cysteine-rich secretory protein (CAP) family were only present in *B. kuwatsukai*, while the cell wall integrity and stress response component (WSC) domain, a putative carbohydrate binding domain was only present in *B. dothidea* (Supplementary Table 11).

Pathogenic and saprophytic fungi can secrete peptidases into their surroundings to degrade a variety of host-related proteases (Wang et al., 2018). This degradation mechanism has potential benefits in eliminating the activity of antifungal host proteins and providing nutrients. Compared with other fungi, *Botryosphaeriaceae* fungi contained a higher number of secretory peptidases. Among all the species, the *M. phaseolina* (*Macrophomina*) fungi contained the largest number of secretory peptidases (*n* = 298), followed by *B. dothidea* (*n* = 264) and *B. kuwatsukai* (*n* = 235), but both were similar to the semi-biotrophic phytopathogenic pathogens *C. higginsianum* (*n* = 233) and *P. oryzae* (*n* = 240). *Diplodia* fungi contain the lowest number of secreted proteins (*n* = 192; *p* = 0.8 × 10^–3^) (Figure 5 and Supplementary Table 10).

##### Carbohydrate Active Enzymes

**FIGURE 6:**
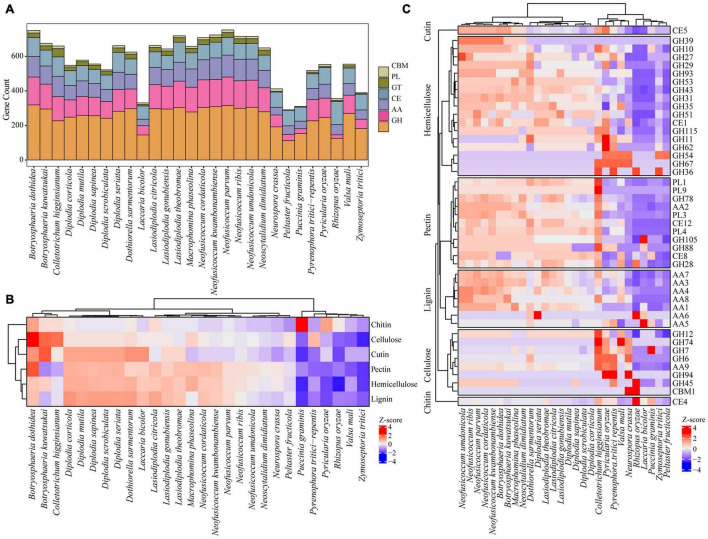
Comparing CAZy among *Botryosphaeriaceae* fungi and other 10 fungal species. **(A)** Six CAZy classes: CBMs, Carbohydrate-Binding Modules; PLs, Polysaccharide Lyases; GTs, Glycosyl Transferases; CEs, Carbohydrate Esterases; AAs, Auxiliary Activities; GHs, Glycoside Hydrolases. **(B)** Distribution of CAZy related to cellulose, hemicellulose and lignin degradation. **(C)** Comparison of selected enzymes involved in PCW (plant cell wall) degradation among *Botryosphaeriaceae* fungi and other 10 fungal species. In heatmaps, Z-score were used to describe over-represented (+ 4–0) and down-represented (0 to −4) gene family.

The ability to degrade complex carbohydrates in plants is an important aspect of the lifestyle of phytopathogenic fungi (Wang et al., 2018). Compared with other species, the fungi in *Botryosphaeriaceae* family contained a higher number of carbohydrate active enzyme-related gene families, such as genes that encode glycoside hydrolases (GHs), polysaccharide lyases (PLs), carbohydrate esterases (CEs), auxiliary activities (AAs), and carbohydrate binding modules (CBMs). Some representative species include *N. parvum* (753), *B. dothidea* (750), *N. kwambonambiense* (724), *L. theobromae* (719), *N. ribis* (716), *N. umdonicola* (716), and *N. cordaticola* (709). Within the *Botryosphaeriaceae* family, *N. parvum* has the largest number of carbohydrate active enzyme-related gene families (753), followed by *B. dothidea* (750), and *B. kuwatsukai* (675). Most *Diplodia* fungi contain fewer carbohydrate activity enzyme-related gene families, such as *D. scrobiculata* (522), *D. corticola* (548), *D. mutila* (577), and *D. sapinea* (556). However, for some species, such as *D. seriata* (662), there was a small increase in the number (Figure 6A and Supplementary Table 12).

The plant cell wall is a complex network structure composed of different polysaccharides, including cellulose, hemicellulose, pectin, and lignin (Terrett and Dupree, 2019). Proteins encoded by fungal carbohydrate active genes and related auxiliary genes can degrade plant cell walls into simple monomers that are absorbed as the carbon source to provide energy for fungi (Islam et al., 2012; Wang et al., 2018). Compared with other fungi, those of *Botryosphaeriaceae* contain expanded gene families involved in the degradation of lignin, cellulose, hemicellulose, and pectin. For example, the gene family involved in the degradation of lignin (107 genes in average) was significantly larger than that in other species (54 genes in average; *p* = 2.0 × 10^–4^, *t*-test). The number of gene families involved in the degradation of hemicellulose and pectin also expanded significantly, with 67 vs. 34 (*p* = 2.0 × 10^–3^) and 70 vs. 32 (*p* = 2.0 × 10^–3^), respectively. Among the *Botryosphaeriaceae* fungi, compared with other genera, it was found that *Neofusicoccum* fungi contain the largest number of gene families involved in the degradation of plant cell walls (287–329), followed by *Botryosphaeria* (219–314) and *Macrophomina* (291–306), while *Diplodia* fungi contain the least number of gene families (215–259) (Figures 6B,C and Supplementary Table 13).

## Discussion

In this study, we conducted a large-scale whole-genome sequencing of 167 *Dothideomycetes* fungi, including 19 species, and comprehensively analyzed evolutionary traits, mating strategies and changes in pathogenic genes in *Dothideomycetes* fungi. Our results can advance our understanding of the evolutionary history of *Botryosphaeriaceae* fungi.

Our results also confirmed that the 167 *Dothideomycetes* fungi can be divided into two subclasses, *Pleosporomycetidae* and *Dothideomycetidae*, and *Botryosphaeriaceae* belongs to *Pleosporomycetidae*, indicating that it is accurate to classify fungi using phylogenies based on phylogenomics (Haridas et al., 2020). Our results inferred the ancestral lifestyle of *Botryosphaeriaceae* fungus as saprobe, and these fungi have undergone at least three transitions from saprophytic to phytopathogenic states. This result is consistent with Schoch et al. (2009). They inferred that the *Dothideomycetes* fungi have experienced multiple transitions from saprophytic pathogens to lichens to phytopathogens, along with multiple transitions from terrestrial to aquatic lifestyles (Schoch et al., 2009). In addition, a larger genome size and a higher number of protein-coding genes are usually associated with saprophytic fungi compared with phytopathogenic fungi. This result is consistent with the findings of Schuelke et al. (2017). They found that in the *Geosmithia* genus, compared with non-pathogenic fungi, the pathogenic fungi in this family have smaller genomes (Schuelke et al., 2017).

Previous studies have shown that phytopathogenic fungi have a smaller genome and contain fewer protein-coding genes compared with saprophytic fungi, which is related to the expansion and contraction of gene families (Ohm et al., 2012; Haridas et al., 2018). In this study, many gene families of phytopathogenic fungi showed contractions, primarily including genes which contain the Pfam domain tetratricopeptide repeat (TPR). TPR can interact with a variety of proteins, such as the anaphase-promoting complex, NADPH oxidases, and HSP90-binding proteins (Das et al., 1998; Kaneko et al., 2006; Kondo et al., 2010). The TPR protein is also part of the plant hormone signaling pathways (Schapire et al., 2006). The family enrichment results found that multiple members of the TRP family proteins (TPR 1, 3, 8, 10, 11, 12, 16, and 19) contracted in phytopathogenic fungi. This contraction may be due to a reduction of signal related TPR proteins in pathogenic fungi. This is because during the process of pathogenic fungal infection, fungal signal related TPR proteins will be affected by plant hormone signals (Haridas et al., 2020). Some other Pfam domains also contracted significantly in phytopathogenic fungi, including PF17111 (the fungal N-terminal domain of STAND proteins), PF05729 (the NACHT domain), and PF14479 (involved in prion inhibition and propagation). These domains are in heterokaryons and play an important role in incompatibility (Paoletti and Saupe, 2009; Greenwald et al., 2010; Daskalov et al., 2012). The contraction of Pfam domains associated with incompatibility in the heterokaryons suggests that other strategies to reduce the level of incompatibility in heterokaryons also exist to improve the adaptability of pathogenic fungi (Ishikawa et al., 2012). Simultaneously, compared with saprophytic fungi, many GO terms are contracted in phytopathogenic fungi, including protein kinases (GO: 0004672), transcription factors (GO: 0003700), zinc ion binding (GO: 0008270), and regulation of nitrogen utilization (GO: 0006808). Given the complexity of amino acid biosynthetic pathways and energy requirements, fungi rely on the absorption of plant amino acids to conserve their own energy. This could be one of the reasons for the contraction of GO terms in plant pathogens (Staats et al., 2013; Mur et al., 2017).

*Botryosphaeriaceae* fungi display both types of mating strategies, homothallism and heterothallism, which are determined by the single locus *MAT1* (Mur et al., 2017). In this study, the mating type genes of 24 *Botryosphaeriaceae* fungal isolates (19 species) were analyzed, and we found that these genes were not highly conserved in terms of their nucleotide and amino acid sequences. However, for two subtype genes, two domains, MATalpha_HMGbox (*MAT1-1-1*) and MATA_HMG-box (*MAT1-2-1*), are more conservative. This is consistent with the fact that the mating type genes of fungi have almost no differences within a species, but they are highly divergent between species (Turgeon, 1998). In many ascomycete fungi, it is a conservative trend that the MATalpha and MATA_HMG-box regions contain introns (Arie et al., 2000; Paoletti et al., 2005; Duong et al., 2013; de miccolis et al., 2016). In *D. sapinea* and *D. seriata*, the introns in the MATA_HMG-box region of the *MAT1-2-1* gene were lost, but the amino acid sequences that flank the lost intron site remain intact (Nagel et al., 2018). This phenomenon is consistent with the intron loss model derived from Poly-A primed mRNA (Sharpton et al., 2008). In this study, with the exception of *Neofusicoccum*, the arrangement of *MAT1-1* and *MAT1-2* genes is highly conserved in six other genera. They all are located between a collinear region that contains four protein-coding genes and one PIM protein, which is the same arrangement as found in most *Phyllostictaceae* fungi (Bihon et al., 2014; Wang et al., 2016). The arrangement of mating type genes of *Neofusicoccum* fungi is exceptional. The locations of the two subtypes of mating type genes are not in conjunction; they are either both located distantly from the collinear region or at different chromosomes (or scaffolds) (Lopes et al., 2017). Similarly, this discontinuous arrangement of mating type genes also exists in *Aspergillus nidulans* (Galagan et al., 2005), *Curvularia cymbopogonis* (Galagan et al., 2005), and *Neosartorya fischeri* (Rydholm et al., 2007).

The mating type gene *MAT1* has two subtypes—*MAT1-1* and *MAT1-2*. When two subtypes are both harbored in the genome, the mating strategy is hom, and if only one is present, it is het (Idnurm, 2011). The repetitive sequence-mediated deletion of one or more mating type genes can cause non-directional changes in mating types, such as the transition from self-fertility to self-sterility (Wilken et al., 2014; Xu et al., 2016; Yun et al., 2017). Such repetitive sequences were not observed in this study. Therefore, *Botryosphaeriaceae* fungi are unlikely to undergo a non-directional change regarding mating types (Nagel et al., 2018). In addition, to further understand the evolutionary characteristics of the fungal mating type in *Botryosphaeriaceae*, we reconstructed the ancestral state of the *Botryosphaeriaceae* fungal mating type and found that the het mating strategy is the ancestral type. Moreover, the fungi in this family experienced a number of transitions to the homothallism strategy, a shift that is common in ascomycete fungi (Inderbitzin et al., 2005; Yokoyama et al., 2006; Nygren et al., 2011; Gioti et al., 2012).

In this study, many gene families of the *Botryosphaeriaceae* fungi have shown significant expansions and contractions, and this change is conducive to the adaptation of fungi to the living environment (Alkan et al., 2013). These contracted gene families include genes that encode secondary metabolite synthases, secreted proteins, and carbohydrate active enzymes. In the family of genes that encode secondary metabolite synthases, *NPRS* and *PKS* gene clusters are responsible for the synthesis of toxic peptides and the production of naphthalenone pentaketides, respectively, in *Botryosphaeriaceae* fungi (*D. seriata*, *L. theobromae* and *N. parvum*) and other pathogenic fungi (*A. fumigatus*, *Diaporthe ampelina*, *Phaeomoniella chlamydospora*, and *Togninia minima*) (Andolfi et al., 2011; Paolinelli-Alfonso et al., 2016). Although the gene clusters of secondary metabolites are not regulated during infection, a large number of products of these gene clusters, which are significantly expanded in the genome, may be involved in the induction of disease symptoms and host adaptation (Yan et al., 2018). Cytochrome P450 is a superfamily of monooxygenases. In addition to participating in the post-synthesis modification of a variety of metabolites, it can also promote the adaptation of fungi to specific ecological niches by altering potentially harmful chemicals in the environment (Siewers et al., 2005). Here, we found the expansion of this gene family, which can explain the wide host range of *Botryosphaeriaceae* fungi. This is because these genes are also associated with some physiological characters; thus, their expansions are likely to promote pathogenic evolution (Nierman et al., 2005). In this study, expansions are obvious for the genes that encode ABC transporters in *Botryosphaeriaceae* fungi, indicating that these fungi have evolved stronger virulence and capacity against plant defense compounds (Han et al., 2001; Luini et al., 2010). Sugar synergistic transporters belong to the MFS family, and they play important roles in fungal spore formation, intercellular communication, and pathogenicity (Gaur et al., 2008). In the *Botryosphaeriaceae* fungi, we found that the sugar synergistic transporter gene family showed different degrees of expansion, which probably makes it more adaptive to different host environments (e.g., different pH values), and more resistant when interacting with various plant pathogens (Zhang et al., 2012; Yin et al., 2015).

Previous studies have showed that secreted proteins play important roles in the infection process of pathogenic fungi (Cobos et al., 2010; O’Connell et al., 2012; Fernandes et al., 2014; Félix et al., 2016, 2019). In this study, the secretory protein gene families of *Botryosphaeriaceae* fungi have expanded to different degrees, but there are large differences between different genera, which may be related to the different infection ranges of *Botryosphaeriaceae* fungi (Wang et al., 2018). In addition, the gene families of carbohydrate active enzymes in *Botryosphaeriaceae* fungi also showed different degrees of expansion. Among these families, the glycoside hydrolase family GH33 is composed of sialidase, which can hydrolyze the glycosidic bonds of terminal sialic acid residues in oligosaccharides. Sialidase can function as a pathogenic factor, facilitating the adaption to the host by evading host recognition or inhibiting host defense responses (Alviano et al., 2004). The cell wall of most dicotyledonous plants is composed of approximately 35% pectin. Pectin-degrading enzymes contribute to the degradation of the cell wall. This local degradation of the cell wall is necessary for fungi to enter the plant cytoplasm and replicate in the host tissue (ten Have et al., 1998). Similar to the highly pathogenic *Colletotrichum higginsianum*, *Neofusicoccum*, and *Botryosphaeria* both possess a larger number of genes encoding pectin-degrading enzymes, which may explain the difference in pathogenicity between *Botryosphaeriaceae* fungi (Wang et al., 2018). Many genes encoding cellulase (AA9 and GH12) and hemicellulase (GH31 and GH43) have been significantly expanded in *Neofusicoccum* and *Botryosphaeria* fungi. These expansions may explain the rapid infection and colonization of *Botryosphaeriaceae* fungi in woody plants (Yan et al., 2018).

## Conclusion

In conclusion, we constructed a phylogenetic tree using whole-genome data and clarified the taxonomic position of *Botryosphaeriaceae* in *Dothideomycetes*. Heterothallism is the ancestral mating type of *Botryosphaeriaceae* fungi, and these fungi have undergone at least 3 transitions from heterothallism to homothallism. The host range of *Botryosphaeriaceae* infection is closely related to the changes in the number of pathogenic genes. Our results provide important insights into the evolutionary history, mating strategies and pathogenicity-related genes variation in *Botryosphaeriaceae*.

## Data Availability Statement

The datasets presented in this study can be found in online repositories. The names of the repository/repositories and accession number(s) can be found below: https://zenodo.org/record/5184447#.YRUFTci0yAc; the sequencing data are found at NCBI (PRJNA777748).

## Author Contributions

CY and HL developed the concept of this study and were main contributors to writing the manuscript. CY, YD, QL, and JZ performed all experiments, data analysis, and prepared figures. CY, HL, SC, XX, AL, and XZ contributed to the manuscript edit and review. All authors read and approved the final manuscript.

## Conflict of Interest

The authors declare that the research was conducted in the absence of any commercial or financial relationships that could be construed as a potential conflict of interest.

## Publisher’s Note

All claims expressed in this article are solely those of the authors and do not necessarily represent those of their affiliated organizations, or those of the publisher, the editors and the reviewers. Any product that may be evaluated in this article, or claim that may be made by its manufacturer, is not guaranteed or endorsed by the publisher.

## Footnotes

1
http://www.repeatmasker.org/RepeatModeler/

2
http://www.repeatmasker.org/

3
http://www.mesquiteproject.org/

## Supplementary Material

Supplementary Figure 1 Whole-genome-based phylogenetic tree of 271 species from Dothideomycetes and 8 outgroups. The two subclass *Pleosporomycetidae* and *Dothideomycetidae* were well resolved.

Supplementary Figure 2 Comparison of genome size between pathogen and saprobe fungi.

Supplementary Figure 3 Comparison of gene count between pathogen and saprobe fungi.

Supplementary Figure 4 Reconstruction of ancestral binary character (non-plant pathogen and plant pathogen) of *Dothideomycetes* using Mesquite based on Mk1 model.

Supplementary Figure 5 Reconstruction of ancestral mating type (heterothallism and homothallism) of *Dothideomycetes* using Mesquite based on Mk1 model as heterothallism.

Supplementary Table 1 List of 271 *Dothideomycetes* fungi and 8 outgroups.

Supplementary Table 2 Classification of 19 *Botryosphaeriaceae* fungi.

Supplementary Table 3 Reconstruction of ancestral binary character (non-plant pathogen and plant pathogen) of *Dothideomycetes* using Mesquite based on Mk1 model.

Supplementary Table 4 Pfam and GO enrichment of *Dothideomycetes* fungi.

Supplementary Table 5 Mating type of *Botryosphaeriaceae* fungi.

Supplementary Table 6 Conserved protein domains of *Botryosphaeriaceae* fungi mating type genes.

Supplementary Table 7 Conservation of mating type genes of *Botryosphaeriaceae* fungi.

Supplementary Table 8 Reconstruction of ancestral mating type of *Botryosphaeriaceae* fungi.

Supplementary Table 9 Gene number of backbone and related genes of secondary metabolism of *Botryosphaeriaceae* fungi and 8 outgroups.

Supplementary Table 10 Gene number of secreted proteins of *Botryosphaeriaceae* fungi and 8 outgroups.

Supplementary Table 11 Pfam annotations of secreted proteins of *B. dothidea* and *B. kuwatsukai*.

Supplementary Table 12 Gene number of CAZy of *Botryosphaeriaceae* fungi and 8 outgroups.

Supplementary Table 13 Genes number of CZAy related to cellulose, hemicellulose and lignin degradation of *Botryosphaeriaceae* fungi and 8 outgroups.

## References

[B1] Alkan N. Espeso E. A. Prusky D. (2013). Virulence regulation of phytopathogenic fungi by pH. *Antioxid. Redox. Signal.* 19 1012–1025. 10.1089/ars.2012.5062 23249178

[B2] Alviano D. S. Rodrigues M. L. Almeida C. A. Santos A. L. Couceiro J. N. Soares R. M. (2004). Differential expression of sialylglycoconjugates and sialidase activity in distinct morphological stages of *Fonsecaea pedrosoi*. *Arch. Microbiol.* 181 278–286. 10.1007/s00203-004-0653-9 14767636

[B3] Amselem J. Cuomo C. A. van Kan J. A. Viaud M. Benito E. P. Couloux A. (2011). Genomic analysis of the necrotrophic fungal pathogens *Sclerotinia sclerotiorum* and *Botrytis cinerea*. *PLoS Genetics* 7:e1002230. 10.1371/journal.pgen.1002230 21876677 PMC3158057

[B4] Andolfi A. Mugnai L. Luque J. Surico G. Cimmino A. Evidente A. (2011). Phytotoxins produced by fungi associated with grapevine trunk diseases. *Toxins (Basel)* 3 1569–1605. 10.3390/toxins3121569 22295177 PMC3268457

[B5] Aramaki T. Blanc-Mathieu R. Endo H. Ohkubo K. Kanehisa M. Goto S. (2020). KofamKOALA: KEGG Ortholog assignment based on profile HMM and adaptive score threshold. *Bioinformatics* 36 2251–2252. 10.1093/bioinformatics/btz859 31742321 PMC7141845

[B6] Arie T. Kaneko I. Yoshida T. Noguchi M. Nomura Y. Yamaguchi I. (2000). Mating-type genes from asexual phytopathogenic ascomycetes *Fusarium oxysporum* and *Alternaria alternata*. *Mol. Plant Microbe Interact.* 13 1330–1339. 10.1094/MPMI.2000.13.12.1330 11106025

[B7] Bankevich A. Nurk S. Antipov D. Gurevich A. A. Dvorkin M. Kulikov A. S. (2012). SPAdes: a new genome assembly algorithm and its applications to single-cell sequencing. *J. Comput. Biol.* 19 455–477. 10.1089/cmb.2012.0021 22506599 PMC3342519

[B8] Bihon W. Burgess T. Slippers B. Wingfield M. J. Wingfield B. D. (2012a). High levels of genetic diversity and cryptic recombination is widespread in introduced *Diplodia pinea* populations. *Australas Plant Pathol.* 41 41–46. 10.1007/s13313-011-0086-2

[B9] Bihon W. Slippers B. Burgess T. Wingfield M. J. Wingfield B. D. (2012b). Diverse sources of infection and cryptic recombination revealed in South African *Diplodia pinea* populations. *Fungal Biol.* 116 112–120. 10.1016/j.funbio.2011.10.006 22208606

[B10] Bihon W. Wingfield M. J. Slippers B. Duong T. A. Wingfield B. D. (2014). MAT gene idiomorphs suggest a heterothallic sexual cycle in a predominantly asexual and important pine pathogen. *Fungal Genet. Biol.* 62 55–61. 10.1016/j.fgb.2013.10.013 24220137

[B11] Billiard S. López-Villavicencio M. Hood M. E. Giraud T. (2012). Sex, outcrossing and mating types: unsolved questions in fungi and beyond. *J. Evol. Biol.* 25 1020–1038. 10.1111/j.1420-9101.2012.02495.x 22515640

[B12] Blanco-Ulate B. Rolshausen P. Cantu D. (2013). Draft genome sequence of *Neofusicoccum parvum* isolate UCR-NP2, a fungal vascular pathogen associated with grapevine cankers. *Genome Announc.* 1:e00339-13. 10.1128/genomeA.00339-13 23766404 PMC3707575

[B13] Bolger A. M. Lohse M. Usadel B. (2014). Trimmomatic: a flexible trimmer for Illumina sequence data. *Bioinformatics* 30 2114–2120. 10.1093/bioinformatics/btu170 24695404 PMC4103590

[B14] Bostock R. M. Pye M. F. Roubtsova T. V. (2014). Predisposition in plant disease: exploiting the nexus in abiotic and biotic stress perception and response. *Annu. Rev. Phytopathol.* 52 517–549. 10.1146/annurev-phyto-081211-172902 25001451

[B15] Camacho C. Coulouris G. Avagyan V. Ma N. Papadopoulos J. Bealer K. (2009). BLAST+: architecture and applications. *BMC Bioinformatics* 10:421. 10.1186/1471-2105-10-421 20003500 PMC2803857

[B16] Campbell M. S. Holt C. Moore B. Yandell M. (2014). Genome annotation and curation using MAKER and MAKER-P. *Curr. Protoc. Bioinform.* 48:4.11.1-39. 10.1002/0471250953.bi0411s48 25501943 PMC4286374

[B17] Castresana J. (2000). Selection of conserved blocks from multiple alignments for their use in phylogenetic analysis. *Mol. Biol. Evol.* 17 540–552. 10.1093/oxfordjournals.molbev.a026334 10742046

[B18] Cobos R. Barreiro C. Mateos R. M. Coque J. J. (2010). Cytoplasmic- and extracellular-proteome analysis of *Diplodia seriata*: a phytopathogenic fungus involved in grapevine decline. *Proteome Sci.* 8:46. 10.1186/1477-5956-8-46 20828386 PMC2944164

[B19] Das A. K. Cohen P. W. Barford D. (1998). The structure of the tetratricopeptide repeats of protein phosphatase 5: implications for TPR-mediated protein-protein interactions. *EMBO J.* 17 1192–1199. 10.1093/emboj/17.5.1192 9482716 PMC1170467

[B20] Daskalov A. Paoletti M. Ness F. Saupe S. J. (2012). Genomic clustering and homology between HET-S and the NWD2 STAND protein in various fungal genomes. *PLoS One* 7:e34854. 10.1371/journal.pone.0034854 22493719 PMC3321046

[B21] de miccolis M. Rotolo C. Pollastro S. Faretra F. (2016). Molecular analysis of the mating type (MAT1) locus in strains of the heterothallic ascomycete *Botrytis cinerea*. *Plant Pathol.* 65 1321–1332.

[B22] Duong T. A. de, Beer Z. W. Wingfield B. D. Wingfield M. J. (2013). Characterization of the mating-type genes in *Leptographium procerum* and *Leptographium profanum*. *Fungal Biol.* 117 411–421. 10.1016/j.funbio.2013.04.005 23809651

[B23] Eddy S. R. (2008). A probabilistic model of local sequence alignment that simplifies statistical significance estimation. *PLoS Comput. Biol.* 4:e1000069. 10.1371/journal.pcbi.1000069 18516236 PMC2396288

[B24] Emms D. M. Kelly S. (2015). OrthoFinder: solving fundamental biases in whole genome comparisons dramatically improves orthogroup inference accuracy. *Genome Biol.* 16:157. 10.1186/s13059-015-0721-2 26243257 PMC4531804

[B25] Félix C. Duarte A. S. Vitorino R. Guerreiro A. C. Domingues P. Correia A. C. (2016). Temperature modulates the secretome of the phytopathogenic fungus *Lasiodiplodia theobromae*. *Front. Plant Sci.* 7:1096. 10.3389/fpls.2016.01096 27536303 PMC4971015

[B26] Félix C. Meneses R. Gonçalves M. Tilleman L. Duarte A. S. Jorrín-Novo J. V. (2019). A multi-omics analysis of the grapevine pathogen *Lasiodiplodia theobromae* reveals that temperature affects the expression of virulence- and pathogenicity-related genes. *Sci. Rep.* 9:13144. 10.1038/s41598-019-49551-w 31511626 PMC6739476

[B27] Fernandes I. Alves A. Correia A. Devreese B. Esteves A. C. (2014). Secretome analysis identifies potential virulence factors of *Diplodia corticola*, a fungal pathogen involved in cork oak (*Quercus suber*) decline. *Fungal Biol.* 118 516–523. 10.1016/j.funbio.2014.04.006 24863480

[B28] Galagan J. E. Calvo S. E. Cuomo C. Ma L. J. Wortman J. R. Batzoglou S. (2005). Sequencing of Aspergillus nidulans and comparative analysis with *A. fumigatus* and *A. oryzae*. *Nature* 438 1105–1115. 16372000 10.1038/nature04341

[B29] Garcia J. F. Lawrence D. P. Morales-Cruz A. Travadon R. Minio A. Hernandez-Martinez R. (2021). Phylogenomics of plant-associated *Botryosphaeriaceae species*. *Front. Microbiol.* 12:652802. 10.3389/fmicb.2021.652802 33815343 PMC8012773

[B30] Gaur M. Puri N. Manoharlal R. Rai V. Mukhopadhayay G. Choudhury D. (2008). MFS transportome of the human pathogenic yeast *Candida albicans*. *BMC Genomics* 9:579. 10.1186/1471-2164-9-579 19055746 PMC2636803

[B31] Gioti A. Mushegian A. A. Strandberg R. Stajich J. E. Johannesson H. (2012). Unidirectional evolutionary transitions in fungal mating systems and the role of transposable elements. *Mol. Biol. Evol.* 29 3215–3226. 10.1093/molbev/mss132 22593224

[B32] Greenwald C. J. Kasuga T. Glass N. L. Shaw B. D. Ebbole D. J. Wilkinson H. H. (2010). Temporal and spatial regulation of gene expression during asexual development of *Neurospora crassa*. *Genetics* 186 1217–1230. 10.1534/genetics.110.121780 20876563 PMC2998306

[B33] Guttman D. S. McHardy A. C. Schulze-Lefert P. (2014). Microbial genome-enabled insights into plant-microorganism interactions. *Nat. Rev. Genet.* 15 797–813. 10.1038/nrg3748 25266034

[B34] Han Y. Liu X. Benny U. Kistler H. C. VanEtten H. D. (2001). Genes determining pathogenicity to pea are clustered on a supernumerary chromosome in the fungal plant pathogen *Nectria haematococca*. *Plant J.* 25 305–314. 10.1046/j.1365-313x.2001.00969.x 11208022

[B35] Haridas S. Albert R. Binder M. Bloem J. LaButti K. Salamov A. (2020). 101 Dothideomycetes genomes: a test case for predicting lifestyles and emergence of pathogens. *Stud. Mycol.* 96 141–153. 10.1016/j.simyco.2020.01.003 32206138 PMC7082219

[B36] Haridas S. Salamov A. Grigoriev I. V. (2018). Fungal genome annotation. *Methods Mol. Biol.* 1775 171–184. 10.1007/978-1-4939-7804-5_15 29876818

[B37] Idnurm A. (2011). Sex and speciation: the paradox that non-recombining DNA promotes recombination. *Fungal Biol. Rev.* 25 121–127. 10.1016/j.fbr.2011.07.003 23136582 PMC3489284

[B38] Inderbitzin P. Harkness J. Turgeon B. G. Berbee M. L. (2005). Lateral transfer of mating system in *Stemphylium*. *Proc. Natl. Acad. Sci. U S A.* 102 11390–11395. 10.1073/pnas.0501918102 16055562 PMC1183548

[B39] Ishikawa F. H. Souza E. A. Shoji J. Y. Connolly L. Freitag M. Read N. D. (2012). Heterokaryon incompatibility is suppressed following conidial anastomosis tube fusion in a fungal plant pathogen. *PLoS One* 7:e31175. 10.1371/journal.pone.0031175 22319613 PMC3271119

[B40] Islam M. S. Haque M. S. Islam M. M. Emdad E. M. Halim A. Hossen Q. M. (2012). Tools to kill: genome of one of the most destructive plant pathogenic fungi *Macrophomina phaseolina*. *BMC Genomics* 13:493. 10.1186/1471-2164-13-493 22992219 PMC3477038

[B41] Kaneko A. Umeyama T. Utena-Abe Y. Yamagoe S. Niimi M. Uehara Y. (2006). Tcc1p, a novel protein containing the tetratricopeptide repeat motif, interacts with Tup1p to regulate morphological transition and virulence in *Candida albicans*. *Eukaryot. Cell* 5 1894–1905. 10.1128/EC.00151-06 16998076 PMC1694794

[B42] Katoh K. Misawa K. Kuma K. Miyata T. (2002). MAFFT: a novel method for rapid multiple sequence alignment based on fast Fourier transform. *Nucleic Acids Res.* 30 3059–3066. 10.1093/nar/gkf436 12136088 PMC135756

[B43] Kim K. Kim K. R. Park E. (2005). An infection model of apple white rot based on conidial germination and appressorium formation of *Botryosphaeria dothidea*. *Plant Pathol. J.* 21 322–327. 10.5423/ppj.2005.21.4.322 PMC4755669 26889109

[B44] Kondo Y. Ohara N. Sato K. Yoshimura M. Yukitake H. Naito M. (2010). Tetratricopeptide repeat protein-associated proteins contribute to the virulence of *Porphyromonas gingivalis*. *Infect. Immun.* 78 2846–2856. 10.1128/IAI.01448-09 20351137 PMC2876578

[B45] Landi L. Pollastro S. Rotolo C. Romanazzi G. Faretra F. De Miccolis Angelini R. M. (2020). Draft genomic resources for the brown rot fungal pathogen *Monilinia laxa*. *Mol. Plant Microbe Interact.* 33 145–148. 10.1094/MPMI-08-19-0225-A 31687915

[B46] Lanfear R. Frandsen P. B. Wright A. M. Senfeld T. Calcott B. (2017). PartitionFinder 2: new methods for selecting partitioned models of evolution for molecular and morphological phylogenetic analyses. *Mol. Biol. Evol.* 34 772–773. 10.1093/molbev/msw260 28013191

[B47] Lomsadze A. Burns P. D. Borodovsky M. (2014). Integration of mapped RNA-Seq reads into automatic training of eukaryotic gene finding algorithm. *Nucleic Acids Res.* 42:e119. 10.1093/nar/gku557 24990371 PMC4150757

[B48] Lopes A. Linaldeddu B. T. Phillips A. J. L. Alves A. (2018). Mating type gene analyses in the genus diplodia: from cryptic sex to cryptic species. *Fungal Biol.* 122 629–638. 10.1016/j.funbio.2018.03.012 29880198

[B49] Lopes A. Phillips A. J. Alves A. (2017). Mating type genes in the genus *Neofusicoccum*: mating strategies and usefulness in species delimitation. *Fungal Biol.* 121 394–404. 10.1016/j.funbio.2016.08.011 28317541

[B50] Luini E. Fleurat-Lessard P. Rousseau L. Roblin G. Berjeaud J.-M. (2010). Inhibitory effects of polypeptides secreted by the grapevine pathogens *Phaeomoniella chlamydospora* and *Phaeoacremonium aleophilum* on plant cell activities. *Physiol. Mol. Plant Pathol.* 74 403–411.

[B51] Marçais G. Kingsford C. (2011). A fast, lock-free approach for efficient parallel counting of occurrences of k-mers. *Bioinformatics* 27 764–770. 10.1093/bioinformatics/btr011 21217122 PMC3051319

[B52] Marchler-Bauer A. Derbyshire M. K. Gonzales N. R. Lu S. Chitsaz F. Geer L. Y. (2015). CDD: NCBI’s conserved domain database. *Nucleic Acids Res.* 43 D222–D226. 10.1093/nar/gku1221 25414356 PMC4383992

[B53] Marsberg A. Kemler M. Jami F. Nagel J. H. Postma-Smidt A. Naidoo S. (2017). Botryosphaeria dothidea: a latent pathogen of global importance to woody plant health. *Mol. Plant Pathol.* 18 477–488. 10.1111/mpp.12495 27682468 PMC6638292

[B54] Meile L. Peter J. Puccetti G. Alassimone J. McDonald B. A. Sánchez-Vallet A. (2020). Chromatin dynamics contribute to the spatiotemporal expression pattern of virulence genes in a fungal plant pathogen. *mBio* 11:e02343-20. 10.1128/mBio.02343-20 33024042 PMC7542367

[B55] Miyauchi S. Kiss E. Kuo A. Drula E. Kohler A. Sánchez-García M. (2020). Large-scale genome sequencing of mycorrhizal fungi provides insights into the early evolution of symbiotic traits. *Nat. Commun.* 11:5125. 10.1038/s41467-020-18795-w 33046698 PMC7550596

[B56] Morales-Cruz A. Amrine K. C. Blanco-Ulate B. Lawrence D. P. Travadon R. Rolshausen P. E. (2015). Distinctive expansion of gene families associated with plant cell wall degradation, secondary metabolism, and nutrient uptake in the genomes of grapevine trunk pathogens. *BMC Genomics* 16:469. 10.1186/s12864-015-1624-z 26084502 PMC4472170

[B57] Mur L. A. J. Simpson C. Kumari A. Gupta A. K. Gupta K. J. (2017). Moving nitrogen to the centre of plant defence against pathogens. *Ann. Bot.* 119 703–709. 10.1093/aob/mcw179 27594647 PMC5378193

[B58] Murray M. G. Thompson W. F. (1980). Rapid isolation of high molecular weight plant DNA. *Nucleic Acids Res.* 8 4321–4325. 10.1093/nar/8.19.4321 7433111 PMC324241

[B59] Nagel J. H. Wingfield M. J. Slippers B. (2018). Evolution of the mating types and mating strategies in prominent genera in the *Botryosphaeriaceae*. *Fungal Genet. Biol.* 114 24–33. 10.1016/j.fgb.2018.03.003 29530630

[B60] Nagel J. H. Wingfield M. J. Slippers B. (2021). Increased abundance of secreted hydrolytic enzymes and secondary metabolite gene clusters define the genomes of latent plant pathogens in the *Botryosphaeriaceae*. *BMC Genomics* 22:589. 10.1186/s12864-021-07902-w 34348651 PMC8336260

[B61] Nguyen N. H. Song Z. Bates S. T. Branco S. Tedersoo L. Menke J. (2016). FUNGuild: an open annotation tool for parsing fungal community datasets by ecological guild. *Fungal Ecol.* 20 241–248. 10.1016/j.funeco.2015.06.006

[B62] Nierman W. C. Pain A. Anderson M. J. Wortman J. R. Kim H. S. Arroyo J. (2005). Genomic sequence of the pathogenic and allergenic filamentous fungus *Aspergillus fumigatus*. *Nature* 438 1151–1156. 10.1038/nature04332 16372009

[B63] Nygren K. Strandberg R. Wallberg A. Nabholz B. Gustafsson T. García D. (2011). A comprehensive phylogeny of *Neurospora* reveals a link between reproductive mode and molecular evolution in fungi. *Mol. Phylogenet. Evol.* 59 649–663. 10.1016/j.ympev.2011.03.023 21439389

[B64] O’Connell R. J. Thon M. R. Hacquard S. Amyotte S. G. Kleemann J. Torres M. F. (2012). Lifestyle transitions in plant pathogenic *Colletotrichum fungi* deciphered by genome and transcriptome analyses. *Nat. Genet.* 44 1060–1065. 10.1038/ng.2372 22885923 PMC9754331

[B65] Ohm R. A. Feau N. Henrissat B. Schoch C. L. Horwitz B. A. Barry K. W. (2012). Diverse lifestyles and strategies of plant pathogenesis encoded in the genomes of eighteen *Dothideomycetes fungi*. *PLoS Pathog* 8:e1003037. 10.1371/journal.ppat.1003037 23236275 PMC3516569

[B66] Paoletti M. Saupe S. J. (2009). Fungal incompatibility: evolutionary origin in pathogen defense? *Bioessays* 31 1201–1210. 10.1002/bies.200900085 19795412

[B67] Paoletti M. Rydholm C. Schwier E. U. Anderson M. J. Szakacs G. Lutzoni F. (2005). Evidence for sexuality in the opportunistic fungal pathogen *Aspergillus fumigatus*. *Curr. Biol.* 15 1242–1248. 10.1016/j.cub.2005.05.045 16005299

[B68] Paolinelli-Alfonso M. Villalobos-Escobedo J. M. Rolshausen P. Herrera-Estrella A. Galindo-Sánchez C. López-Hernández J. F. (2016). Global transcriptional analysis suggests *Lasiodiplodia theobromae* pathogenicity factors involved in modulation of grapevine defensive response. *BMC Genomics* 17:615. 10.1186/s12864-016-2952-3 27514986 PMC4981995

[B69] Peter M. Kohler A. Ohm R. A. Kuo A. Krützmann J. Morin E. (2016). Ectomycorrhizal ecology is imprinted in the genome of the dominant symbiotic fungus *Cenococcum geophilum*. *Nat. Commun.* 7:12662. 10.1038/ncomms12662 27601008 PMC5023957

[B70] Phillips A. J. Alves A. Abdollahzadeh J. Slippers B. Wingfield M. J. Groenewald J. Z. (2013). The *Botryosphaeriaceae*: genera and species known from culture. *Stud. Mycol.* 76 51–167. 10.3114/sim0021 24302790 PMC3825232

[B71] Ranallo-Benavidez T. R. Jaron K. S. Schatz M. C. (2020). GenomeScope 2.0 and Smudgeplot for reference-free profiling of polyploid genomes. *Nat. Commun.* 11:1432. 10.1038/s41467-020-14998-3 32188846 PMC7080791

[B72] Ruibal C. Gueidan C. Selbmann L. Gorbushina A. A. Crous P. W. Groenewald J. Z. (2009). Phylogeny of rock-inhabiting fungi related to *Dothideomycetes*. *Stud. Mycol.* 64 123s–133s. 10.3114/sim.2009.64.06 20169026 PMC2816969

[B73] Rydholm C. Dyer P. S. Lutzoni F. (2007). DNA sequence characterization and molecular evolution of MAT1 and MAT2 mating-type loci of the self-compatible ascomycete mold *Neosartorya fischeri*. *Eukaryot. Cell* 6 868–874. 10.1128/EC.00319-06 17384199 PMC1899244

[B74] Schapire A. L. Valpuesta V. Botella M. A. (2006). TPR proteins in plant hormone signaling. *Plant Signal. Behav.* 1 229–230. 10.4161/psb.1.5.3491 19704665 PMC2634123

[B75] Schoch C. L. Crous P. W. Groenewald J. Z. Boehm E. W. Burgess T. I. de Gruyter J. (2009). A class-wide phylogenetic assessment of *Dothideomycetes*. *Stud. Mycol.* 64 1s–15s. 10.3114/sim.2009.64.01 20169021 PMC2816964

[B76] Schuelke T. A. Wu G. Westbrook A. Woeste K. Plachetzki D. C. Broders K. (2017). Comparative genomics of pathogenic and nonpathogenic beetle-vectored fungi in the Genus Geosmithia. *Genome Biol. Evol.* 9 3312–3327. 10.1093/gbe/evx242 29186370 PMC5737690

[B77] Sharpton T. J. Neafsey D. E. Galagan J. E. Taylor J. W. (2008). Mechanisms of intron gain and loss in *Cryptococcus*. *Genome Biol.* 9:R24. 10.1186/gb-2008-9-1-r24 PMC2395259 18234113

[B78] Siewers V. Viaud M. Jimenez-Teja D. Collado I. G. Gronover C. S. Pradier J. M. (2005). Functional analysis of the cytochrome P450 monooxygenase gene bcbot1 of *Botrytis cinerea* indicates that botrydial is a strain-specific virulence factor. *Mol. Plant Microbe Interact.* 18 602–612. 10.1094/MPMI-18-0602 15986930

[B79] Slater G. S. Birney E. (2005). Automated generation of heuristics for biological sequence comparison. *BMC Bioinformatics* 6:31. 10.1186/1471-2105-6-31 15713233 PMC553969

[B80] Slippers B. Wingfield M. J. (2007). Botryosphaeriaceae as endophytes and latent pathogens of woody plants: diversity, ecology and impact. *Fungal Biol. Rev.* 21 90–106.

[B81] Staats C. C. Kmetzsch L. Schrank A. Vainstein M. H. (2013). Fungal zinc metabolism and its connections to virulence. *Front. Cell Infect. Microbiol.* 3:65. 10.3389/fcimb.2013.00065 24133658 PMC3796257

[B82] Stamatakis A. (2014). RAxML version 8: a tool for phylogenetic analysis and post-analysis of large phylogenies. *Bioinformatics* 30 1312–1313. 10.1093/bioinformatics/btu033 24451623 PMC3998144

[B83] Stanke M. Morgenstern B. (2005). AUGUSTUS: a web server for gene prediction in eukaryotes that allows user-defined constraints. *Nucleic Acids Res.* 33 W465–W467. 10.1093/nar/gki458 15980513 PMC1160219

[B84] Sullivan M. J. Petty N. K. Beatson S. A. (2011). Easyfig: a genome comparison visualizer. *Bioinformatics* 27 1009–1010. 10.1093/bioinformatics/btr039 21278367 PMC3065679

[B85] Tang W. Ding Z. Zhou Z. Q. Wang Y. Z. Guo L. Y. (2012). Phylogenetic and pathogenic analyses show that the causal agent of apple ring rot in China is *Botryosphaeria dothidea*. *Plant Dis.* 96 486–496. 10.1094/PDIS-08-11-0635 30727432

[B86] ten Have A. Mulder W. Visser J. van Kan J. A. (1998). The endopolygalacturonase gene Bcpg1 is required for full virulence of *Botrytis cinerea*. *Mol. Plant Microbe Interact.* 11 1009–1016. 10.1094/MPMI.1998.11.10.1009 9768518

[B87] Terrett O. M. Dupree P. (2019). Covalent interactions between lignin and hemicelluloses in plant secondary cell walls. *Curr. Opin. Biotechnol.* 56 97–104. 10.1016/j.copbio.2018.10.010 30423528

[B88] Turgeon B. G. (1998). Application of mating type gene technology to problems in fungal biology. *Annu. Rev. Phytopathol.* 36 115–137. 10.1146/annurev.phyto.36.1.115 15012495

[B89] van der Nest M. A. Bihon W. De Vos L. Naidoo K. Roodt D. Rubagotti E. (2014). Draft genome sequences of *Diplodia sapinea*, *Ceratocystis manginecans*, and *Ceratocystis moniliformis*. *IMA Fungus* 5 135–140. 25083413 10.5598/imafungus.2014.05.01.13 PMC4107891

[B90] Wang B. Liang X. Gleason M. L. Zhang R. Sun G. (2018). Comparative genomics of *Botryosphaeria dothidea* and *B. kuwatsukai*, causal agents of apple ring rot, reveals both species expansion of pathogenicity-related genes and variations in virulence gene content during speciation. *IMA Fungus* 9 243–257. 10.5598/imafungus.2018.09.02.02 30622881 PMC6317582

[B91] Wang B. Liang X. Hao X. Dang H. Hsiang T. Gleason M. L. (2021). Comparison of mitochondrial genomes provides insights into intron dynamics and evolution in *Botryosphaeria dothidea* and *B. kuwatsukai*. *Environ. Microbiol.* 23 5320–5333. 10.1111/1462-2920.15608 34029452

[B92] Wang N. Y. Zhang K. Huguet-Tapia J. C. Rollins J. A. Dewdney M. M. (2016). Mating type and simple sequence repeat markers indicate a clonal population of *Phyllosticta citricarpa* in Florida. *Phytopathology* 106 1300–1310. 10.1094/PHYTO-12-15-0316-R 27348343

[B93] Waterhouse R. M. Seppey M. Simão F. A. Manni M. Ioannidis P. Klioutchnikov G. (2018). BUSCO applications from quality assessments to gene prediction and phylogenomics. *Mol. Biol. Evol.* 35 543–548. 10.1093/molbev/msx319 29220515 PMC5850278

[B94] Wijayawardene N. Hyde K. Rajeshkumar K. C. Hawksworth D. Madrid H. Kirk P. (2017). Notes for genera: *Ascomycota*. *Fungal Divers* 86 1–594.

[B95] Wilken P. M. Steenkamp E. T. Wingfield M. J. de, Beer Z. W. Wingfield B. D. (2014). DNA loss at the *Ceratocystis fimbriata* mating locus results in self-sterility. *PLoS One* 9:e92180. 10.1371/journal.pone.0092180 24651494 PMC3961304

[B96] Xu C. Wang C. Ju L. Zhang R. Biggs A. R. Tanaka E. (2015). Multiple locus genealogies and phenotypic characters reappraise the causal agents of apple ring rot in China. *Fungal Divers* 71 215–231.

[B97] Xu L. Jardini T. M. Chen W. (2016). Direct repeat-mediated DNA deletion of the mating type MAT1-2 genes results in unidirectional mating type switching in *Sclerotinia trifoliorum*. *Sci. Rep.* 6:27083. 10.1038/srep27083 27255676 PMC4891775

[B98] Yan J. Y. Xie Y. Zhang W. Wang Y. Liu J. K. Hyde K. D. (2013). Species of *Botryosphaeriaceae* involved in grapevine dieback in China. *Fungal Divers* 61 221–236. 10.1007/s13225-013-0251-8

[B99] Yan J. Y. Zhao W. S. Chen Z. Xing Q. K. Zhang W. Chethana K. W. T. (2018). Comparative genome and transcriptome analyses reveal adaptations to opportunistic infections in woody plant degrading pathogens of *Botryosphaeriaceae*. *DNA Res.* 25 87–102. 10.1093/dnares/dsx040 29036669 PMC5824938

[B100] Yin Z. Liu H. Li Z. Ke X. Dou D. Gao X. (2015). Genome sequence of *Valsa canker* pathogens uncovers a potential adaptation of colonization of woody bark. *New Phytol.* 208 1202–1216. 10.1111/nph.13544 26137988

[B101] Yokoyama E. Arakawa M. Yamagishi K. Hara A. (2006). Phylogenetic and structural analyses of the mating-type loci in *Clavicipitaceae*. *FEMS Microbiol. Lett.* 264 182–191. 10.1111/j.1574-6968.2006.00447.x 17064371

[B102] Yun S. H. Kim H. K. Lee T. Turgeon B. G. (2017). Self-fertility in *Chromocrea spinulosa* is a consequence of direct repeat-mediated loss of MAT1-2, subsequent imbalance of nuclei differing in mating type, and recognition between unlike nuclei in a common cytoplasm. *PLoS Genet* 13:e1006981. 10.1371/journal.pgen.1006981 28892488 PMC5608430

[B103] Zhang H. Yohe T. Huang L. Entwistle S. Wu P. Yang Z. (2018). dbCAN2: a meta server for automated carbohydrate-active enzyme annotation. *Nucleic Acids Res.* 46 W95–W101. 10.1093/nar/gky418 29771380 PMC6031026

[B104] Zhang Y. Zhang Z. Zhang X. Zhang H. Sun X. Hu C. (2012). CDR4 is the major contributor to azole resistance among four Pdr5p-like ABC transporters in *Neurospora crassa*. *Fungal Biol.* 116 848–854. 10.1016/j.funbio.2012.05.002 22749171

