# Supplementary figures and images for: Comparative Genomics Reveals Evolutionary Traits, Mating Strategies, and Pathogenicity-Related Genes Variation of Botryosphaeriaceae
Source: Front Microbiol. 2022 Feb 23;13:800981. doi: 10.3389/fmicb.2022.800981 (PMC8905617; doi:10.3389/fmicb.2022.800981)

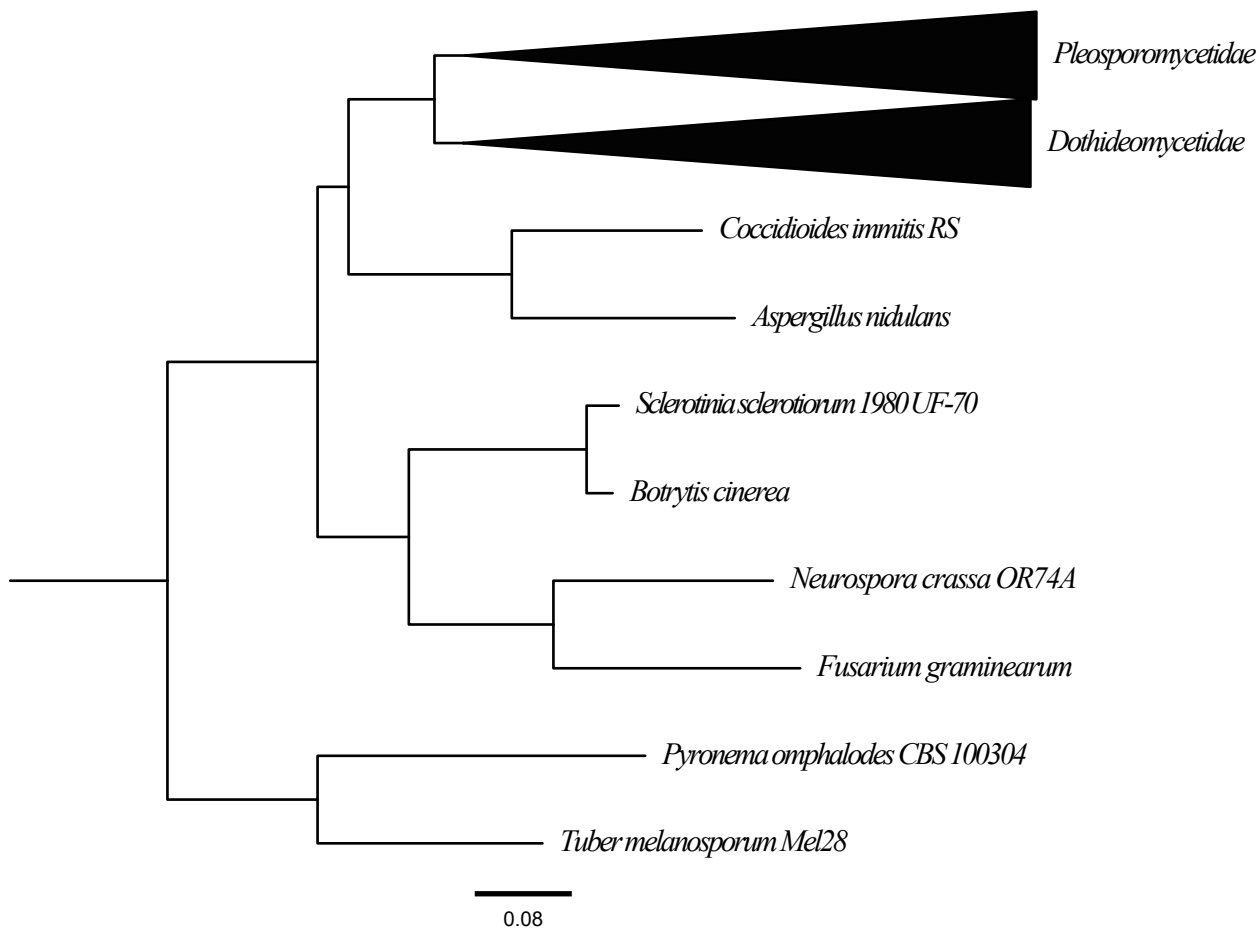

Supplement: Supplementary Figure 1 — Whole-genome-based phylogenetic tree of 271 species from Dothideomycetes and 8 outgroups. The two subclass Pleosporomycetidae and Dothideomycetidae were well resolved. [file Data_Sheet_1.PDF]

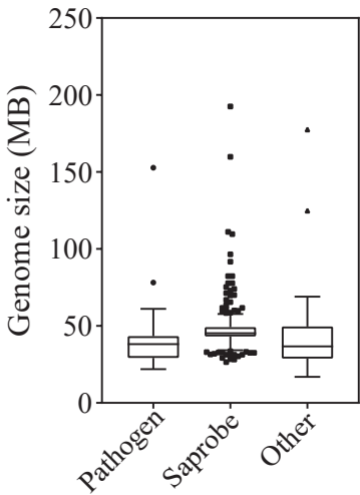

Supplement: Supplementary Figure 2 — Comparison of genome size between pathogen and saprobe fungi. [file Data_Sheet_2.PDF]

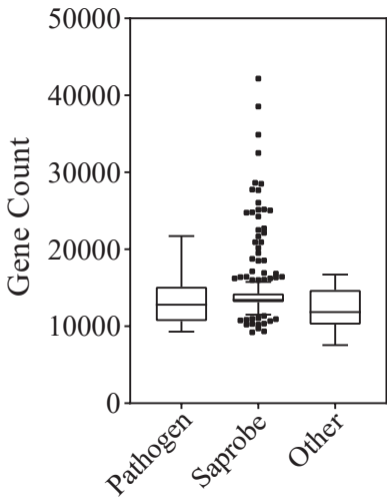

Supplement: Supplementary Figure 3 — Comparison of gene count between pathogen and saprobe fungi. [file Data_Sheet_3.PDF]

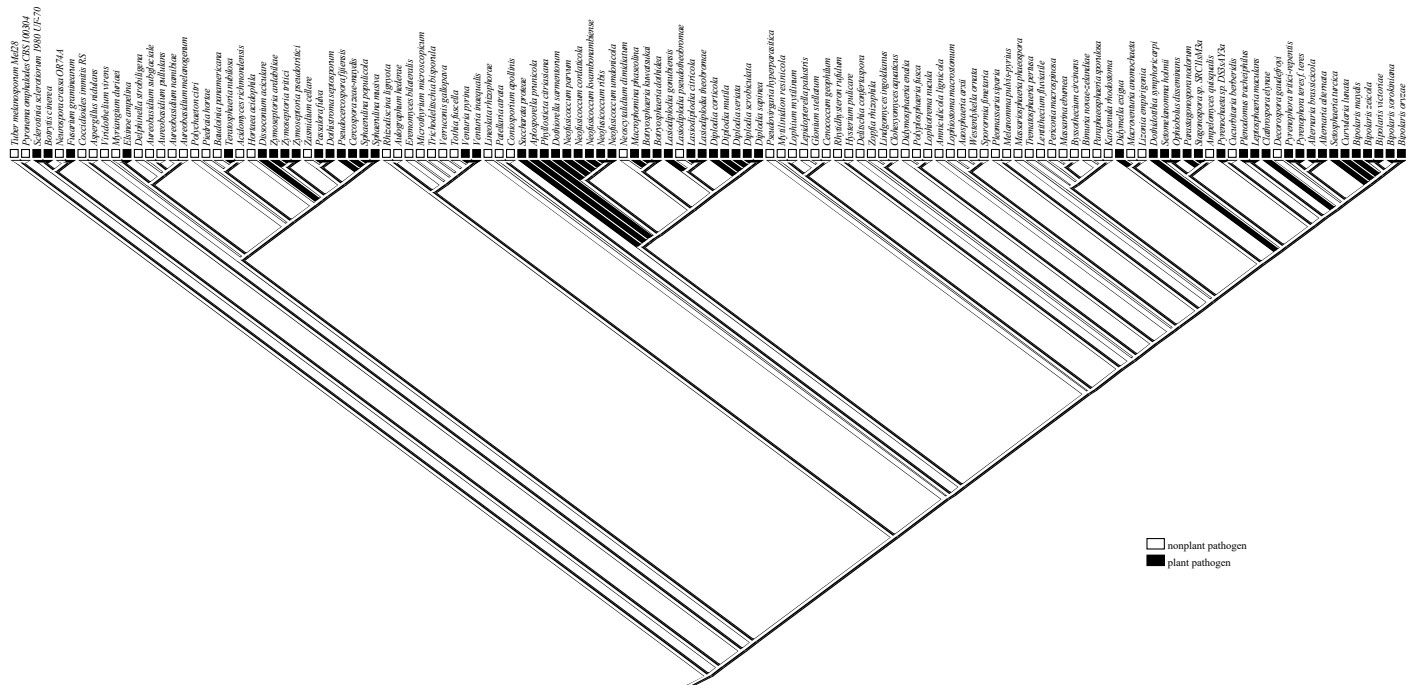

Supplement: Supplementary Figure 4 — Reconstruction of ancestral binary character (non-plant pathogen and plant pathogen) of Dothideomycetes using Mesquite based on Mk1 model. [file Data_Sheet_4.PDF]

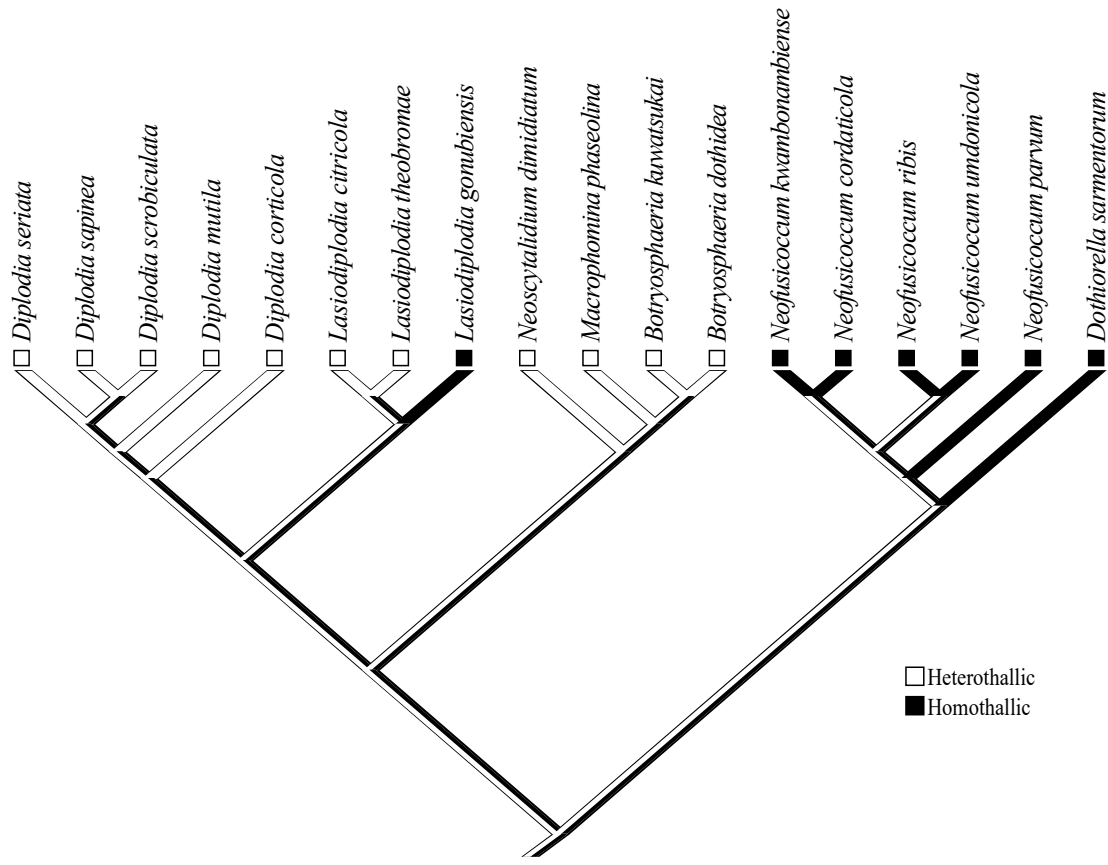

Supplement: Supplementary Figure 5 — Reconstruction of ancestral mating type (heterothallism and homothallism) of Dothideomycetes using Mesquite based on Mk1 model as heterothallism. [file Data_Sheet_5.PDF]
