# Supplementary material for: Comparative Genomics Reveals Evolutionary Traits, Mating Strategies, and Pathogenicity-Related Genes Variation of Botryosphaeriaceae
Source: Front Microbiol. 2022 Feb 23;13:800981. doi: 10.3389/fmicb.2022.800981 (PMC8905617; doi:10.3389/fmicb.2022.800981)
Supplement: Supplementary Table 8 — Reconstruction of ancestral mating type of Botryosphaeriaceae fungi. [file Table_8.pdf]

Tree window for taxa "Taxa"

Showing Tree block: Trees from "Botryosphaeriaceae.18species.aln.phy_phyml_tree.rn.nex"

Original Tree: tree 1 (id 1, version 193)

Tree Description: ((((((Diplodia_seriata:0.004707,(Diplodia_sapinea:0.004044,Diplodia_scrobiculata:0.004059):0.001912):0.016008,Diplodia_mutila:0.016738):0.00643,Diplodia_corticola:0.027671):0.024974,((Lasiodiplodia_citricola:0.003481,Lasiodiplodia_theobromae:0.003572):0.010944,Lasiodiplodia_gonubiensis:0.015881):0.021813):0.038242,(Neoscytalidium_dimidiatum:0.041636,(Macrophomina_phaseolina:0.040666,(Botryosphaeria_kuwatsukai:0.003707,Botryosphaeria_dothidea:0.006891):0.02976):0.011398):0.015593):0.008646,((((Neofusicoccum_kwambonambiense:0.002743,Neofusicoccum_cordaticola:0.002993):0.000248,(Neofusicoccum_ribis:0.001736,Neofusicoccum_umdonicola:0.00104):0.001217):0.000521,Neofusicoccum_parvum:0.003771):0.03777,Dothiorella_sarmentorum:0.081189):0.008646);
Tree with node numbers:

```
                              ,========8 Diplodia seriata
                          ,===7|
                          |   |   ,==10 Diplodia sapinea
                      ,===6|   `===9|
                      |   |       `==11 Diplodia scrobiculata
                  ,===5|   |
                  |   |   `===========12 Diplodia mutila
                  |   |
              ,===4|   `=================13 Diplodia corticola
              |   |
              |   |       ,===========16 Lasiodiplodia citricola
              |   |   ,==15|
          ,===3|   `==14|   `===========17 Lasiodiplodia theobromae
          |   |       |
          |   |       `=================18 Lasiodiplodia gonubiensis
          |   |
          |   |   ,=====================20 Neoscytalidium dimidiatum
          |   `==19|
          |       |   ,=================22 Macrophomina phaseolina
          |       `==21|
          |           |   ,===========24 Botryosphaeria kuwatsukai
======2|              `==23|
          |               `===========25 Botryosphaeria dothidea
          |
          |               ,===========30 Neofusicoccum kwambonambiense
          |           ,==29|
          |           |   `===========31 Neofusicoccum cordaticola
          |       ,==28|
          |       |   |   ,===========33 Neofusicoccum ribis
          |   ,==27|   `==32|
          |   |   |       `===========34 Neofusicoccum umdonicola
          `==26|   |
              |   `=====================35 Neofusicoccum parvum
              |
              `==========================36 Dothiorella sarmentorum
```

Branch lengths

Node	Term/Int	Length
node 2:	Internal	1.7975133655488295E308
node 3:	Internal	0.008646
node 4:	Internal	0.038242
node 5:	Internal	0.024974
node 6:	Internal	0.00643
node 7:	Internal	0.016008
node 8:	Terminal	0.004707
node 9:	Internal	0.001912
node 10:	Terminal	0.004044
node 11:	Terminal	0.004059
node 12:	Terminal	0.016738
node 13:	Terminal	0.027671
node 14:	Internal	0.021813
node 15:	Internal	0.010944
node 16:	Terminal	0.003481
node 17:	Terminal	0.003572
node 18:	Terminal	0.015881
node 19:	Internal	0.015593
node 20:	Terminal	0.041636
node 21:	Internal	0.011398
node 22:	Terminal	0.040666
node 23:	Internal	0.02976
node 24:	Terminal	0.003707
node 25:	Terminal	0.006891
node 26:	Internal	0.008646
node 27:	Internal	0.03777
node 28:	Internal	5.21E-4
node 29:	Internal	2.48E-4
node 30:	Terminal	0.002743
node 31:	Terminal	0.002993
node 32:	Internal	0.001217
node 33:	Terminal	0.001736
node 34:	Terminal	0.00104
node 35:	Terminal	0.003771
node 36:	Terminal	0.081189

--------------- Trace Character History ---------------

Character 1: Character 1
Marginal prob. recon. with model Mk1 (est.) [rate 7.35208959 [est.]] -log L.:6.9994591 (Opt.: width 0.0) Reporting likelihoods as Proportional Likelihoods; Threshold when decisions made: 2 Calc. by Maximum likelihood reconstruct (Generic categorical) (id# 1108)

node 2: Heterothallic: 0.61982036, Homothallic: 0.38017964
node 3: Heterothallic: 0.80714302, Homothallic: 0.19285698
node 4: Heterothallic: 0.90038644*, Homothallic: 0.09961356
node 5: Heterothallic: 0.99163356*, Homothallic: 0.00836644
node 6: Heterothallic: 0.99737034*, Homothallic: 0.00262966
node 7: Heterothallic: 0.99992805*, Homothallic: 0.00007195
node 8: Heterothallic: 1
node 9: Heterothallic: 0.99998327*, Homothallic: 0.00001673
node 10: Heterothallic: 1
node 11: Heterothallic: 1
node 12: Heterothallic: 1
node 13: Heterothallic: 1
node 14: Heterothallic: 0.82912466, Homothallic: 0.17087534
node 15: Heterothallic: 0.99853736*, Homothallic: 0.00146264
node 16: Heterothallic: 1
node 17: Heterothallic: 1
node 18: Homothallic: 1
node 19: Heterothallic: 0.94301493*, Homothallic: 0.05698507
node 20: Heterothallic: 1
node 21: Heterothallic: 0.97059449*, Homothallic: 0.02940551
node 22: Heterothallic: 1
node 23: Heterothallic: 0.99952465*, Homothallic: 0.00047535
node 24: Heterothallic: 1
node 25: Heterothallic: 1
node 26: Heterothallic: 0.43443692, Homothallic: 0.56556308
node 27: Heterothallic: 0.00018754, Homothallic: 0.99981246*
node 28: Heterothallic: 0.00000108, Homothallic: 0.99999892*
node 29: Heterothallic: 0.00000102, Homothallic: 0.99999898*
node 30: Homothallic: 1
node 31: Homothallic: 1
node 32: Heterothallic: 0.00000088, Homothallic: 0.99999912*
node 33: Homothallic: 1
node 34: Homothallic: 1
node 35: Homothallic: 1
node 36: Homothallic: 1
