# Supplementary material for: Comparative Genomics Reveals Evolutionary Traits, Mating Strategies, and Pathogenicity-Related Genes Variation of Botryosphaeriaceae
Source: Front Microbiol. 2022 Feb 23;13:800981. doi: 10.3389/fmicb.2022.800981 (PMC8905617; doi:10.3389/fmicb.2022.800981)
Supplement: Supplementary Table 3 — Reconstruction of ancestral binary character (non-plant pathogen and plant pathogen) of Dothideomycetes using Mesquite based on Mk1 model. [file Table_3.pdf]

Mesquite version 3.61 (build 927)

Tree window for taxa "Taxa"

Showing Tree block: Imported trees

Original Tree: tree 1 (id 1, version 1234)

Tree Description: ((Tuber_melanosporum_Mel28:0.184784,Pyronema_omphalodes_CBS_100304:0.262092):0.126376,((('Sclerotinia sclerotiorum 1980 UF-70':0.024997,Botrytis_cinerea:0.022226):0.14442,(Neurospora_crassa_OR74A:0.183134,Fusarium_graminearum:0.205006):0.120148):0.077337,((Coccidioides_immitis_RS:0.154582,Aspergillus_nidulans:0.185136):0.131301,((Viridothelium_virens:0.227357,(((Myriangium_duriaei:0.114022,Elsinoe_ampelina:0.12453):0.123093,(Delphinella_strobiligena:0.128147,(('Aureobasidium subglaciale ':0.025909,'Aureobasidium pullulans ':0.029722):0.005706,(Aureobasidium_namibiae:0.02405,Aureobasidium_melanogenum:0.02419):0.007508):0.115492):0.053818):0.038813,(Polychaeton_citri:0.176668,((Piedraia_hortae:0.294874,(Baudoinia_panamericana:0.130462,(Teratosphaeria_nubilosa:0.118562,Acidomyces_richmondensis:0.13014):0.021068):0.0071):0.034879,(Hortaea_acidophila:0.175878,(Dissoconium_aciculare:0.173513,(('Zymoseptoria ardabiliae':0.006769,(Zymoseptoria_tritici:0.004563,'Zymoseptoria pseudotritici':0.003675):0.002454):0.12865,((Zasmidium_cellare:0.080428,(Passalora_fulva:0.023299,Dothistroma_septosporum:0.028257):0.089461):0.020818,(Pseudocercospora_fijiensis:0.087433,('Cercospora zeae-maydis':0.06009,(Sphaerulina_populicola:0.008789,Sphaerulina_musiva:0.005604):0.062991):0.069861):0.029454):0.012363):0.023304):0.035111):0.013723):0.027539):0.071996):0.06139):0.022615,(((Rhizodiscina_lignyota:0.187005,Aulographum_hederae:0.214582):0.027644,(Eremomyces_bilateralis:0.274339,(Microthyrium_microscopicum:0.309542,(Trichodelitschia_bisporula:0.191927,(Verruconis_gallopava:0.194225,(Tothia_fuscella:0.136511,(Venturia_pyrina:0.016425,Venturia_inaequalis:0.020976):0.135088):0.029749):0.035143):0.035173):0.045107):0.015553):0.024423,(Lineolata_rhizophorae:0.25002,((Patellaria_atrata:0.178919,Coniosporium_apollinis:0.153189):0.020379,((Saccharata_proteae:0.123549,(Aplosporella_prunicola:0.106041,(Phyllosticta_citriasiana:0.130208,((Dothiorella_sarmentorum_KEdek:0.055749,(Neofusicoccum_parvum:0.001704,(Neofusicoccum_cordaticola:0.001983,(Neofusicoccum_kwambonambiense:0.001506,(Neofusicoccum_ribis:0.001246,Neofusicoccum_umdonicola:0.00065):0.000775):0.000312):0.000756):0.026413):0.010015,((Neoscytalidium_dimidiatum:0.025442,(Macrophomina_phaseolina:0.026383,(Botryosphaeria_kuwatsukai:0.002555,Botryosphaeria_dothidea:0.005159):0.022634):0.009703):0.010964,((Lasiodiplodia_gonubiensis:0.012248,(Lasiodiplodia_pseudotheobromae:0.003848,(Lasiodiplodia_citricola_KE87127:0.001812,'Lasiodiplodia theobromae':0.002405):0.001834):0.006963):0.012571,(Diplodia_corticola:0.0187,(Diplodia_mutila_KE84208:0.01058,(Diplodia_seriata:0.003047,(Diplodia_scrobiculata:0.002789,Diplodia_sapinea:0.002752):0.001559):0.010382):0.004868):0.019805):0.024507):0.006924):0.054302):0.024676):0.029759):0.063087,(Pseudovirgaria_hyperparasitica:0.234491,(((Mytilinidion_resinicola:0.015776,Lophium_mytilinum:0.016322):0.10642,(Lepidopterella_palustris:0.080027,(Glonium_stellatum:0.024867,Cenococcum_geophilum:0.02622):0.043448):0.011133):0.014424,((Rhytidhysteron_rufulum:0.098617,Hysterium_pulicare:0.064345):0.019079,(Delitschia_confertaspora:0.114036,((Zopfia_rhizophila:0.065906,(Lindgomyces_ingoldianus:0.039812,Clohesyomyces_aquaticus:0.065339):0.032652):0.006842,((Didymosphaeria_enalia:0.077903,(Polyplosphaeria_fusca:0.101659,Lophiotrema_nucula:0.075886):0.017304):0.019381,(Amniculicola_lignicola:0.128405,((Lophiostoma_macrostomum:0.093254,(Aaosphaeria_arxii:0.103613,(Westerdykella_ornata:0.074373,Sporormia_fimetaria:0.094832):0.042677):0.011934):0.013077,((Pleomassaria_siparia:0.05681,'Melanomma pulvis-pyrius':0.027465):0.067258,(Massariosphaeria_phaeospora:0.105483,((Trematosphaeria_pertusa:0.057982,(Lentithecium_fluviatile:0.069704,((Periconia_macrospinosa:0.070955,(Massarina_eburnea:0.055303,Byssothecium_circinans:0.039054):0.030382):0.036909,('Bimuria novae-zelandiae':0.042308,(Paraphaeosphaeria_sporulosa:0.014627,Karstenula_rhodostoma:0.017515):0.034048):0.063844):0.013699):0.017877):0.02017,((Didymella_exigua:0.039119,(Macroventuria_anomochaeta:0.01772,Lizonia_empirigonia:0.030969):0.005499):0.070561,(Dothidotthia_symphoricarpi:0.078093,(((Setomelanomma_holmii:0.041223,Ophiobolus_disseminans:0.049557):0.006588,(Parastagonospora_nodorum:0.057256,(Stagonospora_sp.:0.036822,Ampelomyces_quisqualis:0.04176):0.010854):0.011829):0.03405,((Pyrenochaeta_sp.:0.055654,Cucurbitaria_berberidis:0.040598):0.011166,((Plenodomus_tracheiphilus:0.021487,Leptosphaeria_maculans:0.031205):0.04863,(Clathrospora_elynae:0.048148,(Decorospora_gaudefroyi:0.040205,((('Pyrenophora tritici-repentis':0.013049,'Pyrenophora teres':0.006591):0.034873,(Alternaria_brassicicola:0.018852,Alternaria_alternata:0.008881):0.021091):0.005102,(Setosphaeria_turcica:0.029733,(Curvularia_lunata:0.020117,(Bipolaris_maydis:0.005265,(('Bipolaris zeicola':0.000524,Bipolaris_victoriae:0.003756):0.007226,(Bipolaris_sorokiniana:0.006397,Bipolaris_oryzae:0.005549):0.000466):0.004707):0.017765):0.012856):0.024308):0.012964):0.013385):0.016793):0.009691):0.013401):0.014328):0.010348):0.06017):0.013518):0.010682):0.009388):0.008068):0.006457):0.016156):0.028455):0.035808):0.030842):0.050296):0.026926):0.012045):0.011587):0.012637):0.025838):0.068768):0.027079):0.126376);

Tree with node numbers:

```
,==========================247 Tuber melanosporum Mel28
,====3|
|     `==========================248 Pyronema omphalodes CBS 100304
|
|              ,===============6 Sclerotinia sclerotiorum 1980 UF-70
|         ,====5|
|         |     `===============7 Botrytis cinerea
|    ,====4|
|    |    |    ,===============9 Neurospora crassa OR74A
|    |    `====8|
=====2|         `==============10 Fusarium graminearum
     |
     |         ,==============13 Coccidioides immitis RS
     |    ,===12|
     |    |    `==============14 Aspergillus nidulans
     |    |
     |    |    ,=======198 Viridothelium virens
     |    |    |
     |    |    |            ,==202 Myriangium duriaei
     `==246|    |      ,==201|
          |    |      |     `==203 Elsinoe ampelina
          |    |  ,==200|
          |    |  |    |    ,==205 Delphinella strobiligena
          |    |  |    `==204|
          |    |  |         |      ,==207 Aureobasidium subglaciale
          |    |  |         |  ,==206|
          |    |  |         |  |    `==208 Aureobasidium pullulans
          |    |  |         `==212|
          |    |  |            |    ,==210 Aureobasidium namibiae
          |    |  `==199|       `==209|
          |    |                     `==211 Aureobasidium melanogenum
          `===11|
               |   ,==214 Polychaeton citri
               |   |
               |   |      ,==217 Piedraia hortae
               |   `==213|  ,==216|
               |          |  |    |    ,==218 Baudoinia panamericana
               |          |  |    `==222|
               |          |  |          |    ,==219 Teratosphaeria nubilosa
               |          `==215|        `==221|
               |             |               `==220 Acidomyces richmondensis
               |             |    ,==224 Hortaea acidophila
               |             `==223|
               |                  |    ,==226 Dissoconium aciculare
               |                  |    |      ,==229 Zymoseptoria ardabiliae
               |                  `==225|  ,==228|
               |                       |  |    |    ,==230 Zymoseptoria tritici
               |                       |  |    `==232|
               |                       |  |         `==231 Zymoseptoria pseudotritici
               `===15|                  `==227|
                    |                        |    ,==235 Zasmidium cellare
                    |                        |  ,==234|
                    |                        |  |    |    ,==237 Passalora fulva
                    |                        |  |    `==236|
                    |                        `==233|       `==238 Dothistroma septosporum
                    |                             |
                    |                             |    ,==240 Pseudocercospora fijiensis
                    |                             `==239|
                    |                                  |    ,==242 Cercospora zeae-maydis
                    |                                  `==241|
                    |                                       |    ,==244 Sphaerulina populicola
                    |                                       `==243|
                    |                                            `==245 Sphaerulina musiva
                    |
                    |     ,==195 Rhizodiscina lignyota
                    |  ,==181|
                    |  |    `==196 Aulographum hederae
                    |  ,===17|
                    |  |    |  ,==182 Eremomyces bilateralis
                    |  |    `==194|
                    |  |         |  ,==183 Microthyrium microscopicum
                    |  |         `==193|
                    |  |              |  ,==184 Trichodelitschia bisporula
                    |  |              `==192|
                    |  |                   |  ,==185 Verruconis gallopava
                    |  |                   `==191|
                    |  |                        |  ,==186 Tothia fuscella
                    |  |                        `==190|
                    `==197|                          |    ,==187 Venturia pyrina
                       |                             `==189|
                       |                                  `==188 Venturia inaequalis
                       |
                       |  ,==18 Lineolata rhizophorae
                       |  |
                       |  |     ,==177 Patellaria atrata
                       |  |  ,===19|
                       |  |  |    `==178 Coniosporium apollinis
                       |  |  |
                       |  |  |     ,==134 Saccharata proteae
                       |  |  `==180|
                       |  |        |  ,==135 Aplosporella prunicola
                       |  |        `===20|
                       |  |             |  ,==136 Phyllosticta citriasiana
                       |  |             `==175|
                       |  |                  |      ,==163 Dothiorella sarmentorum KEdek
                       |  |                  |  ,==137|
                       |  |                  |  |    |    ,==165 Neofusicoccum parvum
                       |  |                  |  |    `==164|
                       |  |                  `==174|       |    ,==166 Neofusicoccum cordaticola
                       `==179|                     |       `==172|
                          |                        |            |    ,==167 Neofusicoccum kwambonambiense
                          |                        |            `==171|
                          |                        |                 |    ,==168 Neofusicoccum ribis
                          |                        |                 `==170|
                          |                        `==173|                  `==169 Neofusicoccum umdonicola
                          |                             |
                          |                             |       ,==156 Neoscytalidium dimidiatum
                          |                             |  ,==138|
                          |                             |  |    |    ,==158 Macrophomina phaseolina
                          |                             |  |    `==157|
                          |                             |  |         |    ,==159 Botryosphaeria kuwatsukai
                          |                             |  |         `==161|
                          |                             `==162|              `==160 Botryosphaeria dothidea
                          `==176|                            |
                               |                             |       ,==149 Lasiodiplodia gonubiensis
                               |                             |  ,==139|
                               |                             |  |    |    ,==150 Lasiodiplodia pseudotheobromae
                               |                             |  |    `==154|
                               |                             |  |         |    ,==151 Lasiodiplodia citricola KE87127
                               |                             `==155|         `==153|
                               |                                  |              `==152 Lasiodiplodia theobromae
                               |                                  |    ,==140 Diplodia corticola
                               |                                  `==148|
                               |                                       |    ,==141 Diplodia mutila KE84208
                               |                                       `==147|
                               |                                            |    ,==143 Diplodia seriata
                               |                                            `==142|
                               |                                                 |    ,==144 Diplodia scrobiculata
                               |                                                 `==146|
                               |                                                      `==145 Diplodia sapinea
                               |
                               |  ,==21 Pseudovirgaria hyperparasitica
                               |  |
                               |  |      ,==125 Mytilinidion resinicola
                               |  |  ,==124|
                               |  |  |    `==126 Lophium mytilinum
                               `==133|  ,===22|
                                  |  |    |    ,==128 Lepidopterella palustris
                                  |  |    `==127|
                                  |  |         |    ,==130 Glonium stellatum
                                  |  |         `==129|
                                  `==132|              `==131 Cenococcum geophilum
                                     |
                                     |      ,==121 Rhytidhysteron rufulum
                                     |  ,===23|
                                     |  |    `==122 Hysterium pulicare
                                     `==123|
                                        |  ,==24 Delitschia confertaspora
                                        |  |
                                        |  |      ,==115 Zopfia rhizophila
                                        |  |  ,===25|
                                        `==120|  |    |    ,==116 Lindgomyces ingoldianus
                                           |  |    `==118|
                                           |  |         `==117 Clohesyomyces aquaticus
                                           `==119|
                                              |      ,==110 Didymosphaeria enalia
                                              |  ,===26|
                                              |  |    |    ,==111 Polyplosphaeria fusca
                                              |  |    `==113|
                                              |  |         `==112 Lophiotrema nucula
                                              `==114|
                                                 |  ,==27 Amniculicola lignicola
                                                 |  |
                                                 |  |      ,==29 Lophiostoma macrostomum
                                                 |  |  ,===28|
                                                 |  |  |    |  ,==30 Aaosphaeria arxii
                                                 |  |  |    `==34|
                                                 |  |  |       |  ,==31 Westerdykella ornata
                                                 |  |  |       `===33|
                                                 `==109|            `==32 Sporormia fimetaria
                                                    |
                                                    `==108|
                                                       |  ,==106 Pleomassaria siparia
                                                       |  ,===36|
                                                       |  |    `==107 Melanomma pulvis-pyrius
                                                       |  |
                                                       |  |  ,==37 Massariosphaeria phaeospora
                                                       `===35|  |
                                                          |  |    ,==39 Trematosphaeria pertusa
                                                          |  |    |
                                                          |  |  ,===38|  ,==41 Lentithecium fluviatile
                                                          |  |  |    |  |
                                                          |  |  |    |  |    ,==43 Periconia macrospinosa
                                                          `==105|  |    `===40|  ,===42|
                                                             |  |         |  |    |  ,==45 Massarina eburnea
                                                             |  |         |  |    `===44|
                                                             |  |         `===52|       `==46 Byssothecium circinans
                                                             |  |              |
                                                             |  |              |  ,==48 Bimuria novae-zelandiae
                                                             |  |              `===47|
                                                             |  |                   |  ,==49 Paraphaeosphaeria sporulosa
                                                             `==104|                 `===51|
                                                                |                        `==50 Karstenula rhodostoma
                                                                |
                                                                |  ,==100 Didymella exigua
                                                                |  ,===54|
                                                                |  |    |  ,==101 Macroventuria anomochaeta
                                                                |  |    `==103|
                                                                |  |         `==102 Lizonia empirigonia
                                                                `===53|  ,==55 Dothidotthia symphoricarpi
                                                                   |  |
                                                                   |  |      ,==91 Setomelanomma holmii
                                                                   |  |  ,===90|
                                                                   |  |  |    `==92 Ophiobolus disseminans
                                                                   `===99|  ,===56|
                                                                      |  |    |  ,==94 Parastagonospora nodorum
                                                                      |  |    `===93|
                                                                      |  |         |  ,==96 Stagonospora sp.
                                                                      |  |         `===95|
                                                                      `===98|              `==97 Ampelomyces quisqualis
                                                                         |
                                                                         |  ,==87 Pyrenochaeta sp.
                                                                         |  ,===57|
                                                                         |  |    `==88 Cucurbitaria berberidis
                                                                         `===89|
                                                                            |  ,==84 Plenodomus tracheiphilus
                                                                            |  ,===58|
                                                                            |  |    `==85 Leptosphaeria maculans
                                                                            `===86|  ,==59 Clathrospora elynae
                                                                               |  |
                                                                               |  ,==60 Decorospora gaudefroyi
                                                                               `===83|
                                                                                  |  ,==63 Pyrenophora tritici-repentis
                                                                                  |  ,===62|
                                                                                  `===82|  `==64 Pyrenophora teres
                                                                                     |  ,===61|
                                                                                     |  |    |  ,==66 Alternaria brassicicola
                                                                                     |  |    `===65|
                                                                                     `===81|       `==67 Alternaria alternata
                                                                                        |
                                                                                        |  ,==69 Setosphaeria turcica
                                                                                        `===68|  ,==71 Curvularia lunata
                                                                                           |  |
                                                                                           `===70|  ,==73 Bipolaris maydis
                                                                                              |  |
                                                                                              `===72|  ,===75 Bipolaris zeicola
                                                                                                 |  ,===74|
                                                                                                 |  |    `===76 Bipolaris victoriae
                                                                                                 `===80|
                                                                                                    |  ,===78 Bipolaris sorokiniana
                                                                                                    `===77|
                                                                                                       `===79 Bipolaris oryzae
```

Branch lengths

Node	Term/Int	Length
node 2:	Internal	1.7975133655488295E308
node 3:	Internal	0.126376
node 4:	Internal	0.077337
node 5:	Internal	0.14442
node 6:	Terminal	0.024997
node 7:	Terminal	0.022226
node 8:	Internal	0.120148
node 9:	Terminal	0.183134
node 10:	Terminal	0.205006
node 11:	Internal	0.027079
node 12:	Internal	0.131301
node 13:	Terminal	0.154582
node 14:	Terminal	0.185136
node 15:	Internal	0.068768
node 16:	Internal	0.022615
node 17:	Internal	0.024423
node 18:	Terminal	0.25002
node 19:	Internal	0.020379
node 20:	Internal	0.063087
node 21:	Terminal	0.234491
node 22:	Internal	0.014424
node 23:	Internal	0.019079
node 24:	Terminal	0.114036
node 25:	Internal	0.006842
node 26:	Internal	0.019381
node 27:	Terminal	0.128405
node 28:	Internal	0.013077
node 29:	Terminal	0.093254
node 30:	Terminal	0.103613
node 31:	Terminal	0.074373
node 32:	Terminal	0.094832
node 33:	Internal	0.042677
node 34:	Internal	0.011934
node 35:	Internal	0.009388
node 36:	Internal	0.067258
node 37:	Terminal	0.105483
node 38:	Internal	0.02017
node 39:	Terminal	0.057982
node 40:	Internal	0.017877
node 41:	Terminal	0.069704
node 42:	Internal	0.036909
node 43:	Terminal	0.070955
node 44:	Internal	0.030382
node 45:	Terminal	0.055303
node 46:	Terminal	0.039054
node 47:	Internal	0.063844
node 48:	Terminal	0.042308
node 49:	Terminal	0.014627
node 50:	Terminal	0.017515
node 51:	Internal	0.034048
node 52:	Internal	0.013699
node 53:	Internal	0.06017
node 54:	Internal	0.070561
node 55:	Terminal	0.078093
node 56:	Internal	0.03405
node 57:	Internal	0.011166
node 58:	Internal	0.04863
node 59:	Terminal	0.048148
node 60:	Terminal	0.040205
node 61:	Internal	0.005102
node 62:	Internal	0.034873
node 63:	Terminal	0.013049
node 64:	Terminal	0.006591
node 65:	Internal	0.021091
node 66:	Terminal	0.018852
node 67:	Terminal	0.008881
node 68:	Internal	0.024308
node 69:	Terminal	0.029733
node 70:	Internal	0.012856
node 71:	Terminal	0.020117
node 72:	Internal	0.017765
node 73:	Terminal	0.005265
node 74:	Internal	0.007226
node 75:	Terminal	5.24E-4
node 76:	Terminal	0.003756
node 77:	Internal	4.66E-4
node 78:	Terminal	0.006397
node 79:	Terminal	0.005549
node 80:	Internal	0.004707
node 81:	Internal	0.012964
node 82:	Internal	0.013385
node 83:	Internal	0.016793
node 84:	Terminal	0.021487
node 85:	Terminal	0.031205
node 86:	Internal	0.009691
node 87:	Terminal	0.055654
node 88:	Terminal	0.040598
node 89:	Internal	0.013401
node 90:	Internal	0.006588
node 91:	Terminal	0.041223
node 92:	Terminal	0.049557
node 93:	Internal	0.011829
node 94:	Terminal	0.057256
node 95:	Internal	0.010854
node 96:	Terminal	0.036822
node 97:	Terminal	0.04176
node 98:	Internal	0.014328
node 99:	Internal	0.010348
node 100:	Terminal	0.039119
node 101:	Terminal	0.01772
node 102:	Terminal	0.030969
node 103:	Internal	0.005499
node 104:	Internal	0.013518
node 105:	Internal	0.010682
node 106:	Terminal	0.05681
node 107:	Terminal	0.027465
node 108:	Internal	0.008068
node 109:	Internal	0.006457
node 110:	Terminal	0.077903
node 111:	Terminal	0.101659
node 112:	Terminal	0.075886
node 113:	Internal	0.017304
node 114:	Internal	0.016156
node 115:	Terminal	0.065906
node 116:	Terminal	0.039812
node 117:	Terminal	0.065339
node 118:	Internal	0.032652
node 119:	Internal	0.028455
node 120:	Internal	0.035808
node 121:	Terminal	0.098617
node 122:	Terminal	0.064345
node 123:	Internal	0.030842
node 124:	Internal	0.10642
node 125:	Terminal	0.015776
node 126:	Terminal	0.016322
node 127:	Internal	0.011133
node 128:	Terminal	0.080027
node 129:	Internal	0.043448
node 130:	Terminal	0.024867
node 131:	Terminal	0.02622
node 132:	Internal	0.050296
node 133:	Internal	0.026926
node 134:	Terminal	0.123549
node 135:	Terminal	0.106041
node 136:	Terminal	0.130208
node 137:	Internal	0.010015
node 138:	Internal	0.010964
node 139:	Internal	0.012571
node 140:	Terminal	0.0187
node 141:	Terminal	0.01058
node 142:	Internal	0.010382
node 143:	Terminal	0.003047
node 144:	Terminal	0.002789
node 145:	Terminal	0.002752
node 146:	Internal	0.001559
node 147:	Internal	0.004868
node 148:	Internal	0.019805
node 149:	Terminal	0.012248
node 150:	Terminal	0.003848
node 151:	Terminal	0.001812
node 152:	Terminal	0.002405
node 153:	Internal	0.001834
node 154:	Internal	0.006963
node 155:	Internal	0.024507
node 156:	Terminal	0.025442
node 157:	Internal	0.009703
node 158:	Terminal	0.026383
node 159:	Terminal	0.002555
node 160:	Terminal	0.005159
node 161:	Internal	0.022634
node 162:	Internal	0.006924
node 163:	Terminal	0.055749
node 164:	Internal	0.026413
node 165:	Terminal	0.001704
node 166:	Terminal	0.001983
node 167:	Terminal	0.001506
node 168:	Terminal	0.001246
node 169:	Terminal	6.5E-4
node 170:	Internal	7.75E-4
node 171:	Internal	3.12E-4
node 172:	Internal	7.56E-4
node 173:	Internal	0.054302
node 174:	Internal	0.024676
node 175:	Internal	0.029759
node 176:	Internal	0.012045
node 177:	Terminal	0.178919
node 178:	Terminal	0.153189
node 179:	Internal	0.011587
node 180:	Internal	0.012637
node 181:	Internal	0.027644
node 182:	Terminal	0.274339
node 183:	Terminal	0.309542
node 184:	Terminal	0.191927
node 185:	Terminal	0.194225
node 186:	Terminal	0.136511
node 187:	Terminal	0.016425
node 188:	Terminal	0.020976
node 189:	Internal	0.135088
node 190:	Internal	0.029749
node 191:	Internal	0.035143
node 192:	Internal	0.035173
node 193:	Internal	0.045107
node 194:	Internal	0.015553
node 195:	Terminal	0.187005
node 196:	Terminal	0.214582
node 197:	Internal	0.025838
node 198:	Terminal	0.227357
node 199:	Internal	0.06139
node 200:	Internal	0.038813
node 201:	Internal	0.123093
node 202:	Terminal	0.114022
node 203:	Terminal	0.12453
node 204:	Internal	0.053818
node 205:	Terminal	0.128147
node 206:	Internal	0.005706
node 207:	Terminal	0.025909
node 208:	Terminal	0.029722
node 209:	Internal	0.007508
node 210:	Terminal	0.02405
node 211:	Terminal	0.02419
node 212:	Internal	0.115492
node 213:	Internal	0.071996
node 214:	Terminal	0.176668
node 215:	Internal	0.027539
node 216:	Internal	0.034879
node 217:	Terminal	0.294874
node 218:	Terminal	0.130462
node 219:	Terminal	0.118562
node 220:	Terminal	0.13014
node 221:	Internal	0.021068
node 222:	Internal	0.0071
node 223:	Internal	0.013723
node 224:	Terminal	0.175878
node 225:	Internal	0.035111
node 226:	Terminal	0.173513
node 227:	Internal	0.023304
node 228:	Internal	0.12865
node 229:	Terminal	0.006769
node 230:	Terminal	0.004563
node 231:	Terminal	0.003675
node 232:	Internal	0.002454
node 233:	Internal	0.012363
node 234:	Internal	0.020818
node 235:	Terminal	0.080428
node 236:	Internal	0.089461
node 237:	Terminal	0.023299
node 238:	Terminal	0.028257
node 239:	Internal	0.029454
node 240:	Terminal	0.087433
node 241:	Internal	0.069861
node 242:	Terminal	0.06009
node 243:	Internal	0.062991
node 244:	Terminal	0.008789
node 245:	Terminal	0.005604
node 246:	Internal	0.126376
node 247:	Terminal	0.184784
node 248:	Terminal	0.262092

--------------- Trace Character History ---------------

Character 1: npp
Marginal prob. recon. with model Mk1 (est.) [rate 1.59962564 [est.]] -log L.:54.84261106 (Opt.: width 0.0) Reporting likelihoods as Proportional Likelihoods; Threshold when decisions made: 2 Calc. by Maximum likelihood reconstruct (Generic categorical) (id# 1118)

node 2: nonplant pathogen: 0.89316035*, plant pathogen: 0.10683965
node 3: nonplant pathogen: 0.94134698*, plant pathogen: 0.05865302
node 4: nonplant pathogen: 0.60875529, plant pathogen: 0.39124471
node 5: nonplant pathogen: 0.00391131, plant pathogen: 0.99608869*
node 6: plant pathogen: 1
node 7: plant pathogen: 1
node 8: nonplant pathogen: 0.58814293, plant pathogen: 0.41185707
node 9: nonplant pathogen: 1
node 10: plant pathogen: 1
node 11: nonplant pathogen: 0.96133881*, plant pathogen: 0.03866119
node 12: nonplant pathogen: 0.97657503*, plant pathogen: 0.02342497
node 13: nonplant pathogen: 1
node 14: nonplant pathogen: 1
node 15: nonplant pathogen: 0.99830057*, plant pathogen: 0.00169943
node 16: nonplant pathogen: 0.99718361*, plant pathogen: 0.00281639
node 17: nonplant pathogen: 0.99949087*, plant pathogen: 0.00050913
node 18: nonplant pathogen: 1
node 19: nonplant pathogen: 0.99660903*, plant pathogen: 0.00339097
node 20: nonplant pathogen: 0.09844445, plant pathogen: 0.90155555*
node 21: nonplant pathogen: 1
node 22: nonplant pathogen: 0.99985609*, plant pathogen: 0.00014391
node 23: nonplant pathogen: 0.99945421*, plant pathogen: 0.00054579
node 24: nonplant pathogen: 1
node 25: nonplant pathogen: 0.99992329*, plant pathogen: 0.00007671
node 26: nonplant pathogen: 0.99981695*, plant pathogen: 0.00018305
node 27: nonplant pathogen: 1
node 28: nonplant pathogen: 0.99989566*, plant pathogen: 0.00010434
node 29: nonplant pathogen: 1
node 30: nonplant pathogen: 1
node 31: nonplant pathogen: 1
node 32: nonplant pathogen: 1
node 33: nonplant pathogen: 0.99872183*, plant pathogen: 0.00127817
node 34: nonplant pathogen: 0.99968652*, plant pathogen: 0.00031348
node 35: nonplant pathogen: 0.99990489*, plant pathogen: 0.00009511
node 36: nonplant pathogen: 0.99957038*, plant pathogen: 0.00042962
node 37: nonplant pathogen: 1
node 38: nonplant pathogen: 0.99947401*, plant pathogen: 0.00052599
node 39: nonplant pathogen: 1
node 40: nonplant pathogen: 0.99985588*, plant pathogen: 0.00014412
node 41: nonplant pathogen: 1
node 42: nonplant pathogen: 0.99962262*, plant pathogen: 0.00037738
node 43: nonplant pathogen: 1
node 44: nonplant pathogen: 0.99969442*, plant pathogen: 0.00030558
node 45: nonplant pathogen: 1
node 46: nonplant pathogen: 1
node 47: nonplant pathogen: 0.99961502*, plant pathogen: 0.00038498
node 48: nonplant pathogen: 1
node 49: nonplant pathogen: 1
node 50: nonplant pathogen: 1
node 51: nonplant pathogen: 0.99995978*, plant pathogen: 0.00004022
node 52: nonplant pathogen: 0.99981536*, plant pathogen: 0.00018464
node 53: nonplant pathogen: 0.42959419, plant pathogen: 0.57040581
node 54: nonplant pathogen: 0.65448765, plant pathogen: 0.34551235
node 55: plant pathogen: 1
node 56: nonplant pathogen: 0.00029085, plant pathogen: 0.99970915*
node 57: nonplant pathogen: 0.02834726, plant pathogen: 0.97165274*
node 58: nonplant pathogen: 0.00015589, plant pathogen: 0.99984411*
node 59: plant pathogen: 1
node 60: nonplant pathogen: 1
node 61: nonplant pathogen: 0.00004658, plant pathogen: 0.99995342*
node 62: nonplant pathogen: 0.00001244, plant pathogen: 0.99998756*
node 63: plant pathogen: 1
node 64: plant pathogen: 1
node 65: nonplant pathogen: 0.00001503, plant pathogen: 0.99998497*
node 66: plant pathogen: 1
node 67: plant pathogen: 1
node 68: nonplant pathogen: 0.00004384, plant pathogen: 0.99995616*
node 69: plant pathogen: 1
node 70: nonplant pathogen: 0.0000207, plant pathogen: 0.9999793*
node 71: plant pathogen: 1
node 72: nonplant pathogen: 0.00000185, plant pathogen: 0.99999815*
node 73: plant pathogen: 1
node 74: nonplant pathogen: 0.00000006, plant pathogen: 0.99999994*
node 75: plant pathogen: 1
node 76: plant pathogen: 1
node 77: nonplant pathogen: 0.00000008, plant pathogen: 0.99999992*
node 78: plant pathogen: 1
node 79: plant pathogen: 1
node 80: nonplant pathogen: 0.00000008, plant pathogen: 0.99999992*
node 81: nonplant pathogen: 0.00016334, plant pathogen: 0.99983666*
node 82: nonplant pathogen: 0.0081601, plant pathogen: 0.9918399*
node 83: nonplant pathogen: 0.0012492, plant pathogen: 0.9987508*
node 84: plant pathogen: 1
node 85: plant pathogen: 1
node 86: nonplant pathogen: 0.001066, plant pathogen: 0.998934*
node 87: plant pathogen: 1
node 88: nonplant pathogen: 1
node 89: nonplant pathogen: 0.00464909, plant pathogen: 0.99535091*
node 90: nonplant pathogen: 0.00015108, plant pathogen: 0.99984892*
node 91: plant pathogen: 1
node 92: plant pathogen: 1
node 93: nonplant pathogen: 0.00176367, plant pathogen: 0.99823633*
node 94: plant pathogen: 1
node 95: nonplant pathogen: 0.01678489, plant pathogen: 0.98321511*
node 96: plant pathogen: 1
node 97: nonplant pathogen: 1
node 98: nonplant pathogen: 0.00735131, plant pathogen: 0.99264869*
node 99: nonplant pathogen: 0.06931126, plant pathogen: 0.93068874*
node 100: plant pathogen: 1
node 101: nonplant pathogen: 1
node 102: nonplant pathogen: 1
node 103: nonplant pathogen: 0.95247248*, plant pathogen: 0.04752752
node 104: nonplant pathogen: 0.99483114*, plant pathogen: 0.00516886
node 105: nonplant pathogen: 0.99927659*, plant pathogen: 0.00072341
node 106: nonplant pathogen: 1
node 107: nonplant pathogen: 1
node 108: nonplant pathogen: 0.99999168*, plant pathogen: 0.00000832
node 109: nonplant pathogen: 0.99996917*, plant pathogen: 0.00003083
node 110: nonplant pathogen: 1
node 111: nonplant pathogen: 1
node 112: nonplant pathogen: 1
node 113: nonplant pathogen: 0.99938593*, plant pathogen: 0.00061407
node 114: nonplant pathogen: 0.99998712*, plant pathogen: 0.00001288
node 115: nonplant pathogen: 1
node 116: nonplant pathogen: 1
node 117: nonplant pathogen: 1
node 118: nonplant pathogen: 0.99964589*, plant pathogen: 0.00035411
node 119: nonplant pathogen: 0.99997467*, plant pathogen: 0.00002533
node 120: nonplant pathogen: 0.99950537*, plant pathogen: 0.00049463
node 121: nonplant pathogen: 1
node 122: nonplant pathogen: 1
node 123: nonplant pathogen: 0.99983603*, plant pathogen: 0.00016397
node 124: nonplant pathogen: 0.99988843*, plant pathogen: 0.00011157
node 125: nonplant pathogen: 1
node 126: nonplant pathogen: 1
node 127: nonplant pathogen: 0.9997905*, plant pathogen: 0.0002095
node 128: nonplant pathogen: 1
node 129: nonplant pathogen: 0.99987949*, plant pathogen: 0.00012051
node 130: nonplant pathogen: 1
node 131: nonplant pathogen: 1
node 132: nonplant pathogen: 0.99976225*, plant pathogen: 0.00023775
node 133: nonplant pathogen: 0.99306765*, plant pathogen: 0.00693235
node 134: plant pathogen: 1
node 135: plant pathogen: 1
node 136: plant pathogen: 1
node 137: nonplant pathogen: 0.00007671, plant pathogen: 0.99992329*
node 138: nonplant pathogen: 0.00753026, plant pathogen: 0.99246974*
node 139: nonplant pathogen: 0.00020555, plant pathogen: 0.99979445*
node 140: plant pathogen: 1
node 141: plant pathogen: 1
node 142: nonplant pathogen: 0.00000021, plant pathogen: 0.99999979*
node 143: plant pathogen: 1
node 144: plant pathogen: 1
node 145: plant pathogen: 1
node 146: nonplant pathogen: 0.00000005, plant pathogen: 0.99999995*
node 147: nonplant pathogen: 0.00000247, plant pathogen: 0.99999753*
node 148: nonplant pathogen: 0.00000796, plant pathogen: 0.99999204*
node 149: plant pathogen: 1
node 150: nonplant pathogen: 1
node 151: plant pathogen: 1
node 152: plant pathogen: 1
node 153: nonplant pathogen: 0.00002086, plant pathogen: 0.99997914*
node 154: nonplant pathogen: 0.00550013, plant pathogen: 0.99449987*
node 155: nonplant pathogen: 0.00004284, plant pathogen: 0.99995716*
node 156: nonplant pathogen: 1
node 157: nonplant pathogen: 0.0006984, plant pathogen: 0.9993016*
node 158: plant pathogen: 1
node 159: plant pathogen: 1
node 160: plant pathogen: 1
node 161: nonplant pathogen: 0.00000187, plant pathogen: 0.99999813*
node 162: nonplant pathogen: 0.00024584, plant pathogen: 0.99975416*
node 163: plant pathogen: 1
node 164: nonplant pathogen: 0.00000015, plant pathogen: 0.99999985*
node 165: plant pathogen: 1
node 166: plant pathogen: 1
node 167: plant pathogen: 1
node 168: plant pathogen: 1
node 169: plant pathogen: 1
node 170: nonplant pathogen: 2.57164941E-9, plant pathogen: 1*
node 171: nonplant pathogen: 1.50561728E-9, plant pathogen: 1*
node 172: nonplant pathogen: 2.11713853E-9, plant pathogen: 1*
node 173: nonplant pathogen: 0.00008717, plant pathogen: 0.99991283*
node 174: nonplant pathogen: 0.00599686, plant pathogen: 0.99400314*
node 175: nonplant pathogen: 0.01698756, plant pathogen: 0.98301244*
node 176: nonplant pathogen: 0.98594616*, plant pathogen: 0.01405384
node 177: nonplant pathogen: 1
node 178: nonplant pathogen: 1
node 179: nonplant pathogen: 0.99818889*, plant pathogen: 0.00181111
node 180: nonplant pathogen: 0.99922776*, plant pathogen: 0.00077224
node 181: nonplant pathogen: 0.99542987*, plant pathogen: 0.00457013
node 182: nonplant pathogen: 1
node 183: nonplant pathogen: 1
node 184: nonplant pathogen: 1
node 185: nonplant pathogen: 1
node 186: nonplant pathogen: 1
node 187: plant pathogen: 1
node 188: plant pathogen: 1
node 189: nonplant pathogen: 0.00385469, plant pathogen: 0.99614531*
node 190: nonplant pathogen: 0.93172139*, plant pathogen: 0.06827861
node 191: nonplant pathogen: 0.97512596*, plant pathogen: 0.02487404
node 192: nonplant pathogen: 0.99013583*, plant pathogen: 0.00986417
node 193: nonplant pathogen: 0.99381471*, plant pathogen: 0.00618529
node 194: nonplant pathogen: 0.99815775*, plant pathogen: 0.00184225
node 195: nonplant pathogen: 1
node 196: nonplant pathogen: 1
node 197: nonplant pathogen: 0.99978879*, plant pathogen: 0.00021121
node 198: nonplant pathogen: 1
node 199: nonplant pathogen: 0.99439451*, plant pathogen: 0.00560549
node 200: nonplant pathogen: 0.98927847*, plant pathogen: 0.01072153
node 201: nonplant pathogen: 0.8414071, plant pathogen: 0.1585929
node 202: nonplant pathogen: 1
node 203: plant pathogen: 1
node 204: nonplant pathogen: 0.9936442*, plant pathogen: 0.0063558
node 205: nonplant pathogen: 1
node 206: nonplant pathogen: 0.9999763*, plant pathogen: 0.0000237
node 207: nonplant pathogen: 1
node 208: nonplant pathogen: 1
node 209: nonplant pathogen: 0.99997857*, plant pathogen: 0.00002143
node 210: nonplant pathogen: 1
node 211: nonplant pathogen: 1
node 212: nonplant pathogen: 0.99996762*, plant pathogen: 0.00003238
node 213: nonplant pathogen: 0.97840152*, plant pathogen: 0.02159848
node 214: nonplant pathogen: 1
node 215: nonplant pathogen: 0.95622753*, plant pathogen: 0.04377247
node 216: nonplant pathogen: 0.96611502*, plant pathogen: 0.03388498
node 217: nonplant pathogen: 1
node 218: nonplant pathogen: 1
node 219: plant pathogen: 1
node 220: nonplant pathogen: 1
node 221: nonplant pathogen: 0.93199361*, plant pathogen: 0.06800639
node 222: nonplant pathogen: 0.96529991*, plant pathogen: 0.03470009
node 223: nonplant pathogen: 0.88187499*, plant pathogen: 0.11812501
node 224: nonplant pathogen: 1
node 225: nonplant pathogen: 0.18675048, plant pathogen: 0.81324952
node 226: plant pathogen: 1
node 227: nonplant pathogen: 0.06237041, plant pathogen: 0.93762959*
node 228: nonplant pathogen: 0.00002138, plant pathogen: 0.99997862*
node 229: plant pathogen: 1
node 230: plant pathogen: 1
node 231: plant pathogen: 1
node 232: nonplant pathogen: 0.0000004, plant pathogen: 0.9999996*
node 233: nonplant pathogen: 0.05012775, plant pathogen: 0.94987225*
node 234: nonplant pathogen: 0.08293729, plant pathogen: 0.91706271*
node 235: nonplant pathogen: 1
node 236: nonplant pathogen: 0.00118966, plant pathogen: 0.99881034*
node 237: plant pathogen: 1
node 238: plant pathogen: 1
node 239: nonplant pathogen: 0.01392449, plant pathogen: 0.98607551*
node 240: plant pathogen: 1
node 241: nonplant pathogen: 0.0021657, plant pathogen: 0.9978343*
node 242: plant pathogen: 1
node 243: nonplant pathogen: 0.00001534, plant pathogen: 0.99998466*
node 244: plant pathogen: 1
node 245: plant pathogen: 1
node 246: nonplant pathogen: 0.91012211*, plant pathogen: 0.08987789
node 247: nonplant pathogen: 1
node 248: nonplant pathogen: 1
